# The Converging Roles of Nucleases and Helicases in Genome Maintenance and the Aging Process

**DOI:** 10.3390/life15111729

**Published:** 2025-11-10

**Authors:** Aikaterini Margariti, Persefoni Daniil, Theodoros Rampias

**Affiliations:** Biomedical Research Foundation Academy of Athens, 11527 Athens, Greece; katemarg7@gmail.com (A.M.); persadaniil@gmail.com (P.D.)

**Keywords:** helicases, nucleases, genome instability, cellular senescence, DNA damage response (DDR), aging

## Abstract

The process of aging is fundamentally driven by genomic instability and the accumulation of DNA damage, which progressively impair cellular and tissue function. In order to counteract these challenges, cells rely on the DNA damage response (DDR), a multilayered signaling and repair network that preserves genomic integrity and sustains homeostasis. Within this framework, nucleases and helicases have pivotal and complementary roles by remodeling aberrant DNA structures, generating accessible repair intermediates, and determining whether a cell achieves faithful repair, undergoes apoptosis, or enters senescence. Defects in these enzymes are exemplified in human progeroid syndromes, where inherited mutations lead to premature aging phenotypes. This phenomenon is also replicated in genetically engineered mouse models that exhibit tissue degeneration, stem cell exhaustion, and metabolic dysfunction. Beyond their canonical repair functions, helicases and nucleases also interface with the epigenome, as DNA damage-induced chromatin remodeling alters enzyme accessibility, disrupts transcriptional regulation, and drives progressive epigenetic drift and chronic inflammatory signaling. Moreover, their dysfunction accelerates the exhaustion of adult stem cell populations, such as hematopoietic, neural, and mesenchymal stem cells. As a result, tissue regeneration is undermined, establishing a self-perpetuating cycle of senescence, impaired repair, and organismal aging. Current research is focused on developing therapeutic strategies that target the DDR–aging axis on several fronts: by directly modulating repair pathways, by regulating the downstream consequences of senescence, or by preventing DNA damage from accumulating upstream. Taken together, evidence from human disease, animal models, molecular studies, and pharmacological interventions demonstrates that nucleases and helicases are not only essential for genome maintenance but also decisive in shaping aging trajectories. This provides valuable knowledge into how molecular repair pathways influence organismal longevity and age-related diseases.

## 1. DNA Damage: A Fundamental Driver of the Aging Process

Aging is a time-related, multifactorial, degenerative process driven by the progressive accumulation of macromolecular damage [1]. The aging process is attributed to nine major hallmarks: genomic instability, telomere shortening, epigenetic alterations, disrupted intracellular signaling, loss of proteostasis, impaired nutrient regulation, mitochondrial dysfunction, stem cell exhaustion, and cellular senescence [2]. Cellular senescence is a fundamental driver of aging and age-related diseases and can be broadly defined as the irreversible withdrawal of cells from the cell cycle and the loss of their proliferative capacity, due to chronic accumulation of cellular damage [3]. Originally described as a tumor-suppressive mechanism, activated by telomere attrition and DNA damage or oncogenic stress, it is now recognized as having dual implications [4]. While it arrests the proliferation of damaged or potentially malignant cells, it can also lead to tissue dysfunction, aging, and age-related pathologies due to the development of the senescence-associated secretory phenotype (SASP) [5]. SASP consists of cytokines, chemokines, proteases, and growth factors that promote chronic inflammation, fibrosis, and secondary senescence in neighboring cells. The release of these factors can be both beneficial and detrimental: initially, the SASP can aid in tissue repair and immune surveillance, but prolonged SASP activity drives a pro-inflammatory environment that contributes to aging and various diseases, such as osteoarthritis, sarcopenia, and neurodegeneration [6]. Consequently, cellular senescence can function as a safeguard against the accumulation of DNA damage and as a catalyst for tissue dysfunction, thereby amplifying organismal aging.

It is evident that DNA is subject to continuous damage from a variety of endogenous and exogenous sources. These include, firstly, reactive oxygen species (ROS) and their lipid peroxidation products, as well as spontaneous chemical reactions such as hydrolysis and base deamination, together with endogenous alkylation events driven by metabolic methyl donors, all of which continuously generate DNA lesions within the cell [7]. Secondly, environmental agents such as ultraviolet light and genotoxic chemicals have also been identified as contributors to this process. Moreover, the consumption of highly processed foods, which are rich in refined sugars, fats, and chemical additives, can generate ROS and other metabolites that contribute to oxidative stress and DNA damage [8]. Environmental pollution similarly induces DNA damage by creating DNA adducts, strand breaks, and interfering with DNA repair mechanisms, further exacerbating genomic instability [9]. Over time, the persistent accumulation of unrepaired DNA lesions results in the buildup of DNA damage. This triggers the activation of the DNA damage response (DDR), a signaling cascade that maintains cellular homeostasis. The DDR regulates the balance between cell survival and death on the molecular level. To elaborate, DNA damage response halts cell cycle progression and activates various cellular pathways, including those associated with repair, apoptosis, and cellular senescence, thereby determining the cell’s fate [10].

Beyond these immediate cellular outcomes, the long-term persistence or inaccurate repair of DNA lesions fosters the gradual accumulation of mutations that become stably inherited through successive cell generations. This gradual, random process is known as genetic drift. It reflects the random fixation of neutral or nearly neutral variants within somatic cell populations [11]. Acting in parallel with selective pressures, genetic drift progressively reshapes the genomic architecture of tissues, promoting clonal heterogeneity and establishing a substrate for evolutionary adaptation. In aging organisms and developing tumors, such neutral mutational drift generates extensive intratissue diversity, which can subsequently be shaped by selection processes [12]. This underscores a connection between chronic DNA damage and the molecular evolution of cancer, as well as tissue remodeling. In this framework, the DDR not only safeguards the genome acutely but also influences the long-term dynamics of mutation retention and clonal evolution [12,13].

The DDR is organized as a signal-transduction cascade that is often depicted in three tiers. The initial tier includes putative sensors such as the MRN complex, which detect aberrant DNA structures. The second tier consists of central transducers, notably the kinases ATM, ATR, and DNA-PKcs, which amplify and propagate the signal. The third tier is defined by a diverse group of effectors and DNA repair factors. These effectors orchestrate critical processes such as cell-cycle arrest, transcriptional reprogramming, DNA repair, and apoptosis [14]. The restoration of damaged DNA in mammalian cells is dependent on five primary DNA repair pathways: mismatch repair (MMR), base excision repair (BER), nucleotide excision repair (NER), homologous recombination (HR), and non-homologous end joining (NHEJ). BER corrects small base modifications such as oxidation or alkylation, whereas NER removes bulky adducts and UV-induced pyrimidine dimers. MMR resolves replication-associated errors, while double-strand breaks (DSBs) are repaired through HR or NHEJ. These repair mechanisms effectively resolve most DNA lesions during youth, but gradually lose fidelity with age, leading to the accumulation of unrepaired or misrepaired DNA and genomic instability [15].

Within the DDR, nucleases and helicases function as central and complementary effectors. More specifically, they facilitate repair by remodeling damaged DNA and generating accessible intermediates that maintain the substrate in a state suitable for accurate processing. Ultimately, the coordinated action of these enzymes determines whether stalled forks are faithfully rescued or collapse into genome-destabilizing lesions [16]. This review will therefore focus on how DNA damage drives the aging process, with particular attention to the converging roles of nucleases and helicases as critical effectors of genome maintenance.

## 2. Progeroid Syndromes: Human Models of Accelerated Aging from DDR Defects

The correlation between DNA damage and the process of aging is most evident through the study of rare human diseases known as progeroid syndromes. Progeroid syndromes are a group of heterogenous genetic disorders that present a wide range of pathological symptoms [17]. These symptoms are the result of mutations in specific genes that lead to accelerated development of certain characteristics associated with the aging process. As indicated by these symptoms, progeroid syndromes can be classified into two major categories: segmental progeroid syndromes, which affect multiple organs and tissues, and unimodal progeroid syndromes, in which the primary effects are limited to a single organ or tissue [18].

While each syndrome exhibits a distinct spectrum of clinical characteristics, they are all linked to inherited mutations in genes crucial for the maintenance of genomic integrity [19]. In most instances, the affected genes encode proteins that constitute integral components of the DDR. These proteins occupy different functional levels of the DDR network: some act as sensors that recognize DNA lesions, others are helicases that unwind complex DNA structures, while others are nucleases that execute the incisions required for repair pathways. Alternatively, the defect lies in DDR regulators, which are critical for maintaining genome stability and coordinating repair responses. In other cases, the pathogenic mechanism is attributed to the disruption of nuclear envelope integrity, leading to genome destabilization and secondary activation of the DDR [17]. Defects in nuclear envelope components disrupt chromatin organization, gene expression and signaling, contributing to premature cellular senescence [17]. Collectively, these defects lead to persistent DNA damage responses, tissue dysfunction, and accelerated aging. Table 1 summarizes the major human progeroid syndromes, their causative genes and mutations, characteristic manifestations, and the molecular mechanisms linking them to senescence and aging.

As illustrated in the table, the majority of segmental progeroid syndromes are associated with defects in helicases, underscoring their critical functions in genome stability maintenance. In most cases, the underlying mutations are loss-of-function variants, which abolish or severely compromise helicase activity. Disorders such as Xeroderma Pigmentosum, Trichothiodystrophy, Bloom syndrome, and Werner syndrome highlight the pleiotropic roles of helicases, ranging from transcription and DNA repair to recombination and replication, with disruption leading to multisystem premature aging phenotypes [20]. Although nuclease-specific syndromes are rare, nucleases remain equally important, as highlighted by their involvement in Xeroderma Pigmentosum and by disorders of the MRN complex, where precise DNA incision and end-processing are indispensable for genome stability. Beyond these, other DDR-related defects, such as those affecting checkpoint kinases or FA proteins, and abnormalities of the nuclear envelope, as seen in laminopathies, also converge on persistent DNA damage signaling and premature senescence. Taken together, these findings underscore that while helicase dysfunction predominates, both helicases and nucleases occupy a central position in the pathogenesis of human progeroid syndromes. Therefore, supporting that aberrations in these enzymes not only underlie rare monogenic disorders but also mirror universal pathways of ordinary human aging.

## 3. Mouse Models: Experimental Evidence Linking DDR Defects to Aging

Mouse models are among the most significant animal systems for studying aging and age-related human diseases, primarily due to their strong genetic and physiological similarities to humans. The organization and function of major organs and systems is highly conserved, while their relatively short lifespan and ease of breeding make them practical for longitudinal studies [69]. Furthermore, the amenability of mice to genetic manipulation enables the development of a variety of models that mirror specific DDR defects [70]. These models serve as accelerated aging systems that overcome key limitations of natural aging models. Naturally aging mouse models are slow, costly, and prone to individual variability [71], while induced accelerated aging systems offer a more reproducible and cost-effective approach within a shorter timeframe [72].

A wide range of genetically modified mouse models have been developed to investigate how defects in the DDR contribute to cellular senescence and, consequently, accelerate organismal aging. Among these, RecQ helicase-deficient models demonstrate the critical function of these enzymes in stabilizing replication forks and preventing genomic instability [73]. Complementary nuclease-deficient models, reveal that compromised DNA repair mechanisms lead to systemic tissue degeneration and reduced lifespan. Beyond single-gene defects, broader disturbances of DDR signaling reinforce this connection. Mouse models harbouring mutations in checkpoint kinases or FA genes exhibit cumulative DNA damage, chronic DDR activation, and stem cell exhaustion. In parallel, mitochondrial genome instability has been shown to contribute to systemic senescence and premature aging [74]. Similarly, nuclear envelope abnormalities recapitulate human laminopathies, leading to growth retardation, osteoporosis, cardiovascular defects, and early mortality due to disrupted nuclear architecture [75]. A complementary perspective comes from polygenic senescence-accelerated mouse strains (SAMP), which model multifactorial aging processes [76]. Finally, induced accelerated aging systems, such as D-galactose administration or total body irradiation, provide experimentally tractable models of widespread DDR activation, oxidative injury, and senescence induction across multiple organs [77]. Collectively, these models effectively reproduce core aspects of human progeroid syndromes and highlight genome instability as a central driver of aging. The following table (Table 2) summarizes the principal mouse models, their genetic alterations, associated phenotypes, and mechanistic insights.

Taken together, studies of genetically engineered mouse models and accelerated aging systems demonstrate that genome instability is a unifying driver of premature and pathological aging. Whether triggered by helicase dysfunction, nuclease loss, or broader DDR defects, these models consistently show reduced lifespan, stem cell exhaustion, and tissue degeneration that parallel human progeroid syndromes [112]. Importantly, they highlight the complementary and central roles of helicases and nucleases in genome maintenance, whose interplay is essential for replication, recombination, and repair fidelity. Disruption of these functions not only underlies the pathology of rare progeroid disorders but also mirrors the gradual accumulation of senescent phenotypes during normal aging [113].

## 4. Helicases and Nucleases: Divergent Mechanisms, Complementary Functions

The converging evidence from animal models and human progeroid syndromes underscores that helicases and nucleases are indispensable yet mechanistically distinct guardians of genome stability. While both enzyme classes remodel nucleic acids, they do so through fundamentally different biochemical principles. Helicases are ATP-driven molecular motors that unwind or remodel DNA and RNA, whereas nucleases are hydrolytic enzymes that cleave the phosphodiester backbone, usually in a metal-dependent manner [114,115]. These complementary strategies are reflected in their classification, structural architecture, and substrate specificity.

Helicases are universally conserved enzymes that use ATP hydrolysis to translocate along and unwind nucleic acids. At the structural level, all helicases share a conserved ATPase core, related to the bacterial RecA recombinase, which binds and hydrolyzes ATP to generate conformational changes coupled to directional movement. This conserved motor is decorated with additional domains that diversify substrate recognition and protein interactions [116]. On this basis, helicases are broadly classified into six superfamilies (SF1–SF6), distinguished by sequence motifs within the ATPase core and by differences in accessory domains and oligomeric assemblies [117]. Among the six superfamilies, SF1 and SF2 comprise the majority of DNA helicases involved in genome metabolism, with SF2 representing the largest and most structurally diverse group.

Within SF2, several subgroups display distinct structural features and functions. The RecQ family, central to genome integrity, contains helicases with additional RQC and HRDC domains, involved in DNA-structure recognition and protein interactions [118]. WRN, within this subgroup, also possesses a 3′–5′ exonuclease domain [119], while BLM lacks exonuclease activity but participates in Holliday junction dissolution via the BTR complex [120]. RECQL4 a more divergent member of the RecQ family, has a truncated helicase core and a large N-terminal Sld2-like domain essential for replication initiation [120].

A distinct class within SF2 is formed by the Fe–S cluster helicases, characterized by the presence of a [4Fe–4S] cofactor that stabilizes the protein and contributes to the recognition of non-canonical DNA structures. These helicases generally share a RecQ-like helicase motor, containing both Fe–S and ARCH domains. However, they differ in additional domains that define their specific roles. RTEL1 includes a long C-terminal region with domains that mediate PCNA binding and interaction with R-loops and G-quadruplexes [121]. XPB and XPD, on the other hand, are adapted for local DNA unwinding during transcription initiation and lesion recognition in nucleotide excision repair [122]. Lastly, FANCM is unique in using its ATPase motor for branch migration of DNA intermediates, with its C-terminal FAAP24-interacting domain anchoring it within the Fanconi anemia pathway, highlighting its specialized DNA remodeling role [123].

**Figure 1:**
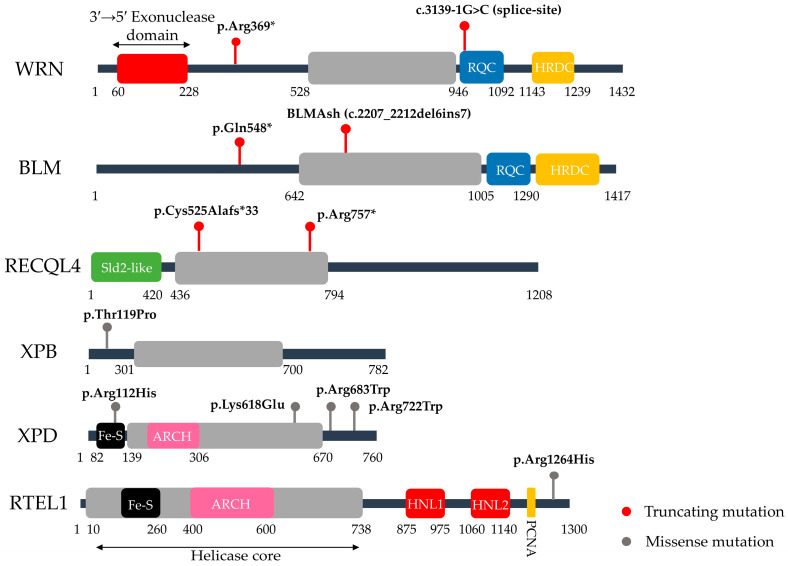
Domain architecture and recurrent pathogenic variants in SF2 helicases. Schematic representation of conserved domains and recurrent pathogenic variants in RecQ and Fe–S helicases. The RecQ family (WRN, BLM, RECQL4) shares a helicase core flanked by RQC and HRDC domains, with WRN uniquely containing an N-terminal exonuclease and RECQL4 an N-terminal Sld2-like region essential for replication initiation. The Fe–S helicases (XPB, XPD, RTEL1) feature a [4Fe–4S] cofactor and ARCH domain, while RTEL1 additionally includes C-terminal harmonin-like (HNL1/HNL2) and PCNA-binding motifs that maintain replication-fork stability and telomere integrity. The mutations shown represent the most frequent pathogenic variants associated with Werner syndrome (WRN), Bloom syndrome (BLM), Rothmund–Thomson syndrome (RECQL4), and the transcription- and repair-related disorders Trichothiodystrophy (XPD, XPB) and Xeroderma Pigmentosum (XPD), as well as Dyskeratosis Congenita (RTEL1). Truncating mutations (red; nonsense, frameshift, or splice-site) remove essential catalytic regions, while missense variants (grey) cluster within helicase or Fe–S cores, impairing ATPase activity or protein interactions critical for genome maintenance.

The significance of these domains is highlighted by the clustering of pathogenic variants associated with major human progeroid syndromes within the conserved helicase cores and accessory domains of RecQ and Fe–S helicases (Table 1). In WRN, recurrent variants such as p.Arg369* or splice-site mutations truncate the protein before or within the helicase core, abolishing ATPase and exonuclease functions and explaining the classical Werner phenotype [24]. In BLM, founder mutations like BLMAsh map directly to the RecQ helicase motor, impairing Holliday junction dissolution and driving the hyper-recombination seen in Bloom syndrome [29]. Similarly, RECQL4 frameshift and nonsense mutations frequently localize to the helicase domain or its N-terminal extension essential for replication initiation, consistent with the genomic instability and developmental defects of Rothmund–Thomson syndrome [34]. In the Fe–S helicases XPB (ERCC3) and XPD (ERCC2), recurrent missense mutations fall within the helicase core or the TFIIH-interacting interfaces, explaining the spectrum of transcription and repair defects underlying Xeroderma Pigmentosum (XP) and Trichothiodystrophy (TTD) [38,42]. Likewise, in the Fe–S helicase RTEL1, missense variants such as p.Arg1264H is impair T-loop disassembly, leading to defective telomere replication and the critically short telomeres that underlie Dyskeratosis Congenita (DC). Together, these mutational hotspots demonstrate that the catalytic cores and interaction modules of helicases are the most frequent targets in human disease, reinforcing their indispensability for genome maintenance (Figure 1).

Nucleases, unlike helicases, are classified by the type of cleavage they perform and by the structural fold of their catalytic domains. The fundamental division is between endonucleases, which cut within DNA strands, and exonucleases, which degrade DNA from free termini. At the structural level, most DNA repair nucleases belong to three major families: the RNase H-like fold, the PD-(D/E)XK family, and the phosphodiesterase family, with some hybrid enzymes combining multiple features [115].

RNase H-like nucleases include well-defined examples from NER. The ERCC1–XPF heterodimer is a central case, where XPF carries the catalytic nuclease domain, while ERCC1 stabilizes DNA substrates. XPF also contains a helicase-like domain for substrate recognition [124]. XPG, another RNase H-like enzyme, works as a monomer and uses a DNA-binding groove to recognize bubble structures for precise incision, with its N-terminal catalytic region separated by a spacer [125].

The PD-(D/E)XK family exemplifies exonucleolytic activity. Members of this family share a conserved catalytic motif (PD-(D/E)XK), in which acidic residues coordinate metal ions to catalyze phosphodiester bond hydrolysis. EXO1 is a key representative, with an extended processivity domain enabling 5′–3′ resection over long distances, regulated by protein partners to prevent uncontrolled degradation [126]. In contrast to the PD-(D/E)XK fold, the phosphodiesterase family has a distinct fold, often assembling as dimers and coordinating metal cofactors via histidine and aspartate. A prominent member is MRE11, which forms a dimer with a dual-metal active site, enabling both endonucleolytic cleavage and exonucleolytic resection at double-strand breaks. Its dimeric structure also plays a role in the MRN complex, linking enzymatic activity with scaffolding functions [127]. Finally, hybrid architectures are seen in DNA2, which combines nuclease and helicase activities. Despite containing both domains, its biological function is dominated by the nuclease activity, with helicase activity playing a secondary role in DNA flap processing during extensive DNA end resection [128].

**Figure 2:**
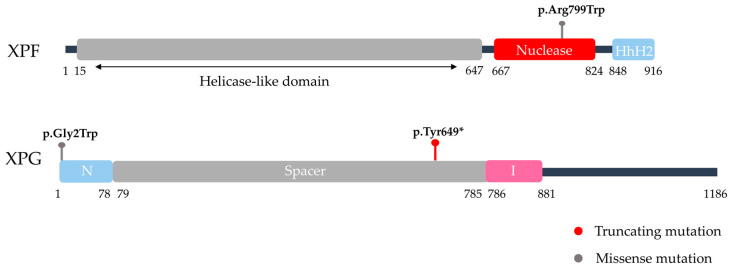
Domain architecture and recurrent pathogenic variants in RNase H-like nucleases. Schematic of conserved domains and recurrent disease-associated variants in XPF (ERCC4) and XPG (ERCC5). Both endonucleases operate in nucleotide excision repair, with XPF functioning as part of the ERCC1–XPF heterodimer and XPG acting as a monomeric nuclease. Truncating mutations (red) remove essential C-terminal regions required for TFIIH interaction, while missense variants (grey) within the catalytic RNase H-like fold impair incision activity, leading to Xeroderma Pigmentosum (XP) or combined XP/Cockayne syndrome (XP/CS) phenotypes.

The importance of nuclease domains is also underscored by the recurrent pathogenic mutations with major human progeroid syndromes listed in Table 1. In XPF (ERCC4), recurrent variants such as p.Arg799Trp affect the nuclease catalytic fold and adjacent DNA-binding motifs, weakening ERCC1 binding and incision activity on bubble substrates. These defects underlie the excision-repair impairment seen in Xeroderma Pigmentosum (XP), while more severe disruptions have been linked to combined XP/Cockayne syndrome (XP/CS) presentations [39]. In XPG (ERCC5), recurrent changes including p.Gly2Trp and p.Tyr649* affect either the catalytic N-terminal core or truncate the C-terminal region, respectively. Missense variants in the catalytic core abolish incision activity and result in classical XP, as reflected in Table 1, whereas truncating mutations that delete the C-terminal interaction domains disrupt TFIIH contacts and, in some instances, lead to the combined XP/CS phenotype [39]. Collectively, these examples show that nuclease-related disorders arise from mutations striking the very domains essential for catalysis and protein interactions, reinforcing their central role in genome maintenance (Figure 2).

The comparison of these enzyme classes underscores two contrasting biochemical strategies for DNA processing. Helicases are ATP-driven molecular motors, structurally versatile and often conformationally flexible, that remodel nucleic acids through translocation and unwinding. Nucleases, in contrast, are hydrolytic enzymes with rigid catalytic scaffolds that cleave the phosphodiester backbone with high spatial precision. While their mechanisms are distinct, both strategies exemplify how evolution has generated complementary solutions to the common challenge of maintaining DNA structure. This structural and biochemical contrast provides the framework for considering helicases and nucleases as interconnected components of the genome maintenance network.

## 5. The Genome Maintenance Network: An Interplay of Helicases and Nucleases

The genome maintenance network represents a dynamic ensemble of enzymes that collectively safeguard genetic stability under conditions of replication stress and DNA damage. Central to this network are two classes of factors with complementary and interdependent functions. Nucleases act as molecular scissors that cleave DNA, thereby enabling the removal of obstructive structures and the processing of DNA intermediates. Helicases function as molecular motors that unwind double-stranded DNA, dismantle secondary structures, and remodel stalled intermediates [129,130]. Together, these enzyme families establish the backbone of the cellular response to replication-associated stress.

DNA replication represents one of the most vulnerable phases of the cell cycle, as the progression of replication forks is constantly challenged by impediments such as bulky DNA lesions, tightly bound protein–DNA complexes, repetitive elements, or conflicts between replication and transcription [131]. These obstacles can lead to the formation of stalled replication forks, which constitute one of the most frequent endogenous threats to genome integrity. Stalled forks are highly dynamic structures that require stabilization and regulated processing to avoid collapse into DSBs [132].

Once a fork has stalled, several distinct outcomes are possible depending on the cellular context and the availability of fork protection factors. One possible outcome is fork stabilization. In this case, RPA rapidly coats the exposed ssDNA, while BRCA1/2 and RAD51 protect the nascent strands from nucleolytic degradation [133,134]. Checkpoint activation through ATR–CHK1 halts cell cycle progression, giving the cell time to respond [135]. The stalled fork is thus maintained in an intact, protected state until the blocking lesion is removed by the appropriate DNA repair pathway. Another possible response is fork reversal, in which the fork is remodeled into a four-way Holliday junction-like intermediate; this structural rearrangement provides time for lesion repair and prevents fork breakage [134]. Alternatively, the fork may undergo limited nucleolytic resection followed by homologous recombination (HR), where enzymes such as MRE11 and EXO1 generate substrates for RAD51-mediated strand invasion and fork restart. Finally, replication may resume through damage-bypass mechanisms, including translation synthesis (TLS) or template switching, which allow the fork to progress across or around the lesion without extensive remodeling [133,134].

**Figure 3:**
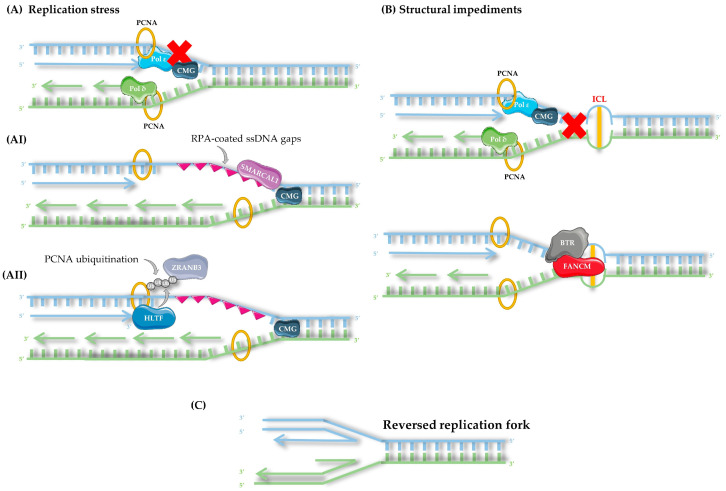
Context-dependent mechanisms of replication fork reversal. (**A**) Replication stress (e.g., nucleotide depletion or protein–DNA obstacles) results in uncoupling of the replicative helicase (CMG) from the polymerase. (**AI**) SMARCAL1 translocase is recruited to RPA-coated single-stranded DNA (ssDNA) gaps, promoting fork remodeling. (**AII**) HLTF translocase promotes PCNA polyubiquitination and, along with ZRANB3 translocase, further facilitates fork reversal by driving regression and recognizing polyubiquitinated PCNA. (**B**) Structural impediments. Stalled forks at interstrand crosslinks (ICLs) are recognized by FANCM helicase/translocase, which, in coordination with the BTR complex, catalyzes branch migration and regression into a non-crossover pathway. (**C**) Reversed fork stabilization: Both replication stress- and structure-induced pathways converge on the formation of a four-way Holliday junction-like intermediate.

Among the possible outcomes of fork stalling, fork reversal has emerged as one of the most significant responses to replication stress, as it directly influences genome stability, repair pathway choice, and checkpoint activation. When fork reversal occurs, the stalled fork is remodeled into a four-way Holliday junction-like structure [136,137]. Formation of this junction relies on a coordinated but context-dependent set of enzymes. When the block is primarily sensed through replication stress signals, SNF2-family translocases provide the motor activity. SMARCAL1 translocase is recruited to forks with RPA-coated ssDNA gaps, ZRANB3 translocase recognizes polyubiquitinated PCNA, and HLTF translocase both promotes PCNA ubiquitination and drives reversal through its HIRAN domain [137,138]. By contrast, when the impediment reflects structural challenges within the DNA itself, helicases contribute in a context-dependent manner. FANCM helicase/translocase collaborates with the FA core complex to catalyze branch migration and directs fork regression into a non-crossover pathway via the BLM–TOP3A–RMI1/2 (BTR) complex [51]. BLM and WRN helicases act at fragile loci, such as telomeres and G-quadruplexes, facilitating fork regression and secondary structure resolution [135,138]. RAD51, along with BRCA1 and BRCA2, stabilizes regressed forks, protecting nascent strands from nucleolytic degradation by MRE11, DNA2, and EXO1 [132] (Figure 3).

From this reversed fork state, several possible outcomes can follow. In the first pathway, nucleases are recruited to process aberrant DNA intermediates. The MRN complex introduces short-range resection at regressed arms, with MRE11 functioning as a nuclease, RAD50 as an ATPase that tethers the ends, and NBS1 as a regulatory subunit [139]. The nuclease DNA2, often acting in cooperation with RecQ helicases such as WRN, degrades displaced 5′ nascent strands, while EXO1 provides long-range processive resection when restart requires extensive end processing [140]. Under normal conditions, these activities promote fork restart and lesion bypass [141]. In the absence of protective mechanisms, uncontrolled nuclease activity causes extensive DNA strand degradation, leading to chromosomal instability [128]. To counteract this, RAD51 forms nucleoprotein filaments on the single-stranded DNA during fork reversal, shielding nascent strands from degradation by MRE11 or DNA2 [142]. This protection relies on tumor suppressors BRCA1 and BRCA2 [143]. When BRCA2 is absent, this protection fails, leaving nascent DNA vulnerable to nucleolytic attack and increasing the risk of fork collapse. Under normal conditions, stabilized RAD51 filaments promote strand invasion into the sister chromatid, thereby enabling homologous recombination and faithful restart of replication [135].

In the second pathway, restoration of the replication fork following reversal occurs independently of homologous recombination. Instead of engaging the RAD51-mediated strand invasion machinery, the reversed fork is directly remodeled back to its original configuration through the action of branch migration enzymes. Two key players in this conservative mechanism are the FANCM translocase and the RECQ1 helicase [144]. FANCM translocase facilitates fork remodelling and prevents error-prone repair, such as break-induced replication, which could cause genomic instability [145]. RECQ1 also aids fork restoration without extensive resection, promoting a recombination-independent recovery mechanism that ensures replication fidelity [118]. Together, these enzymes support a streamlined, recombination-independent recovery mechanism that bypasses the lesion and restores replication fidelity.

**Figure 4:**
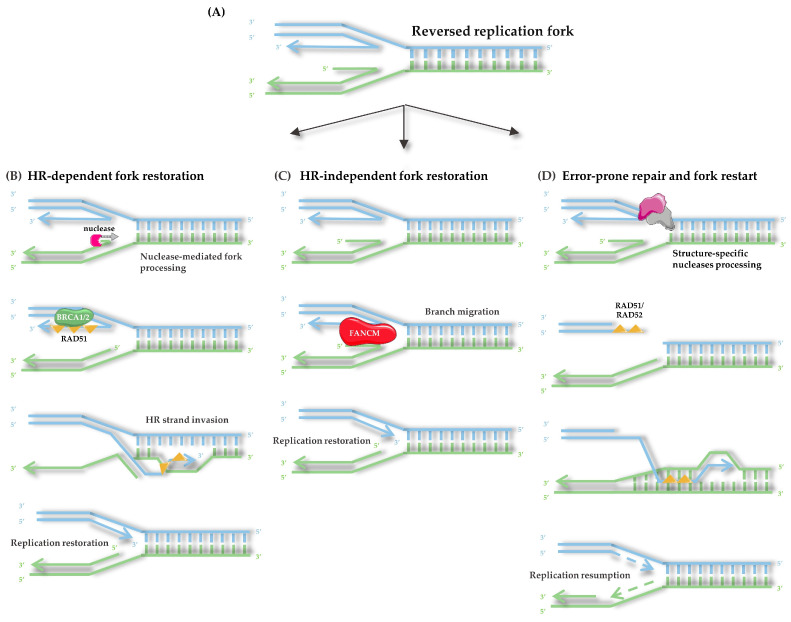
Context-dependent outcomes of replication fork reversal. (**A**) Reversal of a replication fork generates a four-way junction, which can be processed through several distinct pathways. (**B**) HR-dependent fork restoration: in the first pathway, nucleases (e.g., DNA2 and EXO1) process aberrant DNA intermediates. RAD51 filaments, stabilized by BRCA1/2 and accessory proteins, protect nascent strands from excessive degradation. When stabilized, RAD51 filaments promote homologous recombination (HR) and accurate replication resumption. (**C**) HR-independent fork restoration: in the second pathway, homologous recombination is not required. Translocases such as FANCM catalyze branch migration, restoring the fork to its original configuration once the blocking lesion is bypassed. (**D**) Error-prone repair and fork restart: in the third pathway, when fork restoration fails, structure-specific nucleases (e.g., MUS81–EME1 or GEN1) cleave the junction, producing a one-ended double-strand break. RAD51 or RAD52, depending on the cell cycle timing, mediate strand invasion at the exposed 3′ end, facilitating fork restart.

In the third pathway, when a reversed replication fork cannot be properly restored, either due to persistent obstacles, disrupted remodeling, or failure of protective mechanisms, the fork structure is resolved through endonucleolytic cleavage. Structure-specific nucleases such as MUS81–EME1 and GEN1 recognize and cleave the Holliday junction-like intermediates formed during fork reversal, resulting in the formation of a one-ended DSB [146]. In S/G2, RAD51 filaments assemble on the exposed 3′ end and mediate strand invasion into the sister chromatid, followed by long-range DNA synthesis through break-induced replication (BIR). If the lesion persists into mitosis, a similar reaction occurs but is mediated primarily by RAD52, which promotes mitotic DNA synthesis (MiDAS) at under-replicated fragile regions [147]. Although these processes are more error-prone than branch migration or BRCA-dependent fork protection, they provide an essential salvage pathway that ensures completion of DNA replication under persistent stress [148] (Figure 4).

The dynamic interplay of helicases, nucleases, and recombinases at stalled forks exemplifies the modular nature of the genome maintenance network. When this interplay is maintained, stalled forks can resume DNA synthesis through recombination-mediated restart or template switching. When it fails, however, stalled forks collapse into DSBs, one of the most cytotoxic forms of genomic damage [130,149]. When a DSB occurs, the cell must choose between NHEJ, a rapid but error-prone process, or HR, a high-fidelity pathway that relies on the coordinated action of nucleases, helicases, and recombinases, together with their regulatory cofactors [150].

Initiation of HR requires controlled DNA end resection. The MRN complex (MRE11–RAD50–NBS1) recognizes the break. Together with the endonuclease CtIP, MRN introduces short-range incisions that remove protein–DNA obstacles and prepare the DNA ends for subsequent processing [151]. These initial cuts are not sufficient on their own but serve as a licensing step that enables the recruitment of nucleases and helicases responsible for long-range resection.

**Figure 5:**
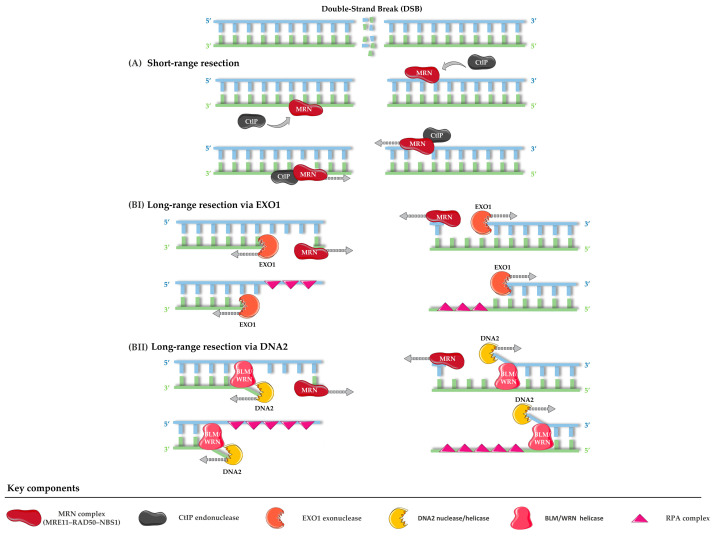
Initiation and extension of DNA end resection at double-strand breaks. (**A**) The MRN complex (MRE11–RAD50–NBS1) together with CtIP recognizes DNA double-strand breaks and initiates short-range resection. MRE11 provides 3′ → 5′ exonuclease activity and, in cooperation with CtIP, introduces the initial incisions that license long-range processing. (**BI**) Long-range resection pathway mediated by EXO1 exonuclease, which digests the 5′ strand in the 5′ → 3′ direction, generating 3′ single-stranded DNA regions that are rapidly coated by RPA. (**BII**) Alternative long-range resection pathway in which DNA2 nuclease collaborates with RecQ helicases (BLM/WRN); the helicase unwinds the duplex while DNA2 cleaves the displaced 5′ strand in the 5′ → 3′ direction, with RPA binding to protect the exposed ssDNA.

Long-range resection is then carried out by two partially redundant pathways. In the first, the exonuclease EXO1 digests the 5′ strand in a processive manner. In the second, the nuclease DNA2 collaborates with RecQ family helicases such as BLM or WRN, where the helicase unwinds the duplex and DNA2 cleaves the displaced 5′ strand. This helicase–nuclease collaboration ensures efficient formation of extended 3′ ssDNA tails, which serve as substrates for recombinase loading [129,152]. These extended ssDNA regions must be carefully controlled: insufficient resection prevents HR from proceeding, whereas excessive processing can destabilize the genome. To maintain this balance, BRCA1 stimulates both EXO1 and the DNA2–helicase pathway, while checkpoint signaling through ATR limits EXO1 activity to avoid uncontrolled degradation [150]. Once resection has been properly regulated, the resulting ssDNA becomes the substrate for the next stage of HR, beginning with RPA binding (Figure 5).

RPA, the main single-stranded DNA-binding protein in eukaryotes, rapidly coats the resected DNA. By binding with high affinity, it protects the ssDNA from nucleolytic degradation and prevents the formation of secondary structures that could block repair [153]. However, this protective role also creates a kinetic barrier for recombination, since RPA-covered DNA cannot be readily accessed by RAD51 recombinase [149]. To resolve this conflict, cells employ multiple strategies. The best-characterized is the mediator pathway, where the BRCA1–PALB2–BRCA2 axis replaces RPA with RAD51, enabling filament assembly and homology search [154]. In cases of inefficient handover or increased replication stress, helicases like HELQ collaborate with RAD51 or RPA to direct repair towards error-prone pathways, such as SSA or MMEJ, affecting the repair fidelity [155].

Once RAD51 has displaced RPA, the presynaptic filament initiates homology search and promotes strand invasion, generating a D-loop structure. The choice between two repair pathways, synthesis-dependent strand annealing (SDSA) and double-strand break repair (DSBR), is influenced by helicases such as RTEL1, BLM, RECQ5, and FBH1, which destabilize D-loops, promoting SDSA. In the absence of these helicases, D-loops are more stable, favouring DSBR and the formation of double Holliday junctions (dHJs) [156].

In mitotic cells, dHJs are typically resolved by the BTR complex (BLM helicase, TOP3A topoisomerase, and RMI1/2), leading to non-crossover products [157]. If dissolution proves insufficient, structure-specific nucleases such as MUS81–EME1 or GEN1, coordinated by the SLX4 scaffold, resolve dHJs, which can produce crossover products. This nuclease-dependent resolution is more error-prone, but necessary for completing repair [158]. Finally, the repair process concludes with gap filling by DNA polymerases (primarily Pol δ and Pol ε) and the sealing by DNA ligase I, restoring intact chromosomes [159].

As evident from the mechanisms mentioned, nucleases and helicases play essential roles in maintaining genome integrity, particularly under replication stress and DNA damage. These enzymes are vital components of the cellular DNA repair machinery, working together to ensure that DNA lesions are properly recognized, processed, and repaired to prevent mutations and chromosomal instability. Their dysfunction or failure can impair the ability of cells to effectively respond to DNA damage, leading to the accumulation of unrepaired or misrepaired DNA lesions. Over time, this accumulation compromises the stability of the genome, contributing to cellular aging and dysfunction. This is particularly evident in human progeroid syndromes, which serve as clear models of how defects in nucleases and helicases can accelerate the aging process. These findings highlight the critical roles of nucleases and helicases in maintaining genome stability, as well as their potential impact on the aging process.

## 6. Epigenome–Genome Crosstalk: Helicases and Nucleases at the Chromatin Interface

Genome maintenance does not occur in isolation but within the highly organized context of chromatin. DNA is wrapped around nucleosomes and further shaped by DNA methylation, histone modifications, and remodeling complexes. These epigenetic features create a dynamic environment that influences how accessible damaged sites are, how repair proteins are recruited, and how efficiently repair proceeds [160,161]. In this way, genome stability is tightly coupled to the state of the epigenome.

Within this chromatin context, the activity of helicases and nucleases is affected by epigenetic features. DNA methylation, histone modifications, and nucleosome positioning determine whether these enzymes can be efficiently recruited to damaged sites. Compact chromatin generally restricts their action, while more open configurations facilitate processing and repair, although certain enzymes can still function within heterochromatic domains [162]. In this way, the epigenome sets the stage on which genome maintenance occurs. If this regulation is disrupted, repair becomes inefficient, damage accumulates, and chromatin itself undergoes progressive disorganization. Such failures contribute to genome instability and are increasingly observed in aging cells, where enzymatic activity is compromised alongside epigenetic drift [163].

DNA methylation is one of the most fundamental epigenetic modifications, involving the addition of a methyl group to cytosine residues within CpG dinucleotides. This modification shapes chromatin architecture by recruiting methyl-binding proteins and histone modifiers, thereby restricting DNA accessibility and reinforcing heterochromatin [164]. Within this methylated and often compact chromatin, helicases continue to function as key guardians of genome stability. Members of the RecQ family, including WRN and BLM, are particularly important because they unwind structured DNA elements and stabilize replication forks in heterochromatic regions, where compaction would otherwise stall replication [165]. By contrast, in more open chromatin, helicases like FANCJ and RTEL1 prevent fork stalling by resolving G-quadruplexes and other secondary structures at CpG-rich promoters, showing that their function spans both compact and relaxed environments depending on context. Nucleases, by contrast, depend on a more relaxed chromatin state to carry out their processing activities. The MRN complex, through the nuclease function of MRE11, is less effective in densely methylated regions, while EXO1 achieves long-range resection more efficiently when chromatin is accessible. The impact of DNA methylation on genome stability is therefore twofold: it regulates how repair enzymes such as helicases and nucleases can act, and it also shapes transcriptional dynamics at CpG-rich regions. In this transcriptional context, CpG regions are particularly prone to R-loop formation, where an RNA transcript hybridizes with the DNA template and leaves the opposite strand single-stranded [165]. If unresolved by helicases such as BLM or SETX, these R-loops become substrates for nucleases including XPF and XPG, generating DNA breaks and thereby linking transcriptional regulation to genome instability [166].

Histone post-translational modifications (PTMs) represent another major layer of epigenetic regulation. These chemical changes include acetylation, methylation, phosphorylation, and ubiquitination of histone tails, each of which alters chromatin compaction and repair factor recruitment [167]. Acetylation marks, such as H4K16ac, generally relax nucleosome–nucleosome interactions, creating an open chromatin state. This facilitates the access of nucleases, allowing XPF–ERCC1 to efficiently unhook interstrand crosslinks or MUS81 to cleave stalled replication intermediates. In contrast, repressive marks such as H3K9me3 or macroH2A promote chromatin compaction and restrict factor binding [168]. In these contexts, helicases like FANCJ and RTEL1 are required to unwind secondary DNA structures embedded in heterochromatin, thereby preventing persistent fork stalling and facilitating repair. Through this interplay, histone modifications function as signals that determine whether helicases or nucleases can act efficiently at a given site.

Telomeres provide a clear example of how histone modifications intersect with DNA repair in a specialized chromatin domain. Chromosome ends are packaged into heterochromatin enriched in H3K9me3, H4K20me3, and DNA methylation, and further protected by the shelterin complex [169]. This configuration represses inappropriate repair events that would otherwise fuse chromosome ends. Within this restrictive environment, helicases such as RTEL1, WRN, and BLM dismantle G-quadruplexes and recombination intermediates that accumulate during replication [170]. At the same time, nucleases like Apollo and EXO1 process the natural DNA overhangs at telomeric ends, ensuring their proper replication and capping [171]. When these mechanisms fail, telomeres lose their protective structure, become fragile, and trigger DNA damage responses, illustrating how defects in helicase or nuclease function at telomeric chromatin directly compromise genome stability [172].

Chromatin remodeling represents another critical layer of epigenome–genome crosstalk. Remodelers use ATP hydrolysis to reposition or restructure nucleosomes, thereby regulating DNA accessibility without directly cutting or unwinding DNA. For example, SWI/SNF complexes mobilize nucleosomes to generate a more open chromatin configuration. This allows helicases such as WRN and BLM to resolve structured DNA and permits nucleases like MRE11 and EXO1 to initiate resection at DNA breaks [173]. A specialized example is ATRX, a remodeler of the SWI/SNF-like helicase/ATPase family, which deposits the histone variant H3.3 at heterochromatin and telomeres, suppressing G-quadruplex formation and reducing unscheduled nuclease cleavage [174]. When remodeling is defective, nucleosomes remain improperly positioned, creating barriers for replication and repair. This is particularly detrimental at fragile sites and telomeres, where remodeler activity is essential to prevent persistent replication stress and safeguard genome stability.

The breakdown of helicase–nuclease coordination within the chromatin environment has consequences that extend far beyond inefficient repair. Persistent DNA damage and unresolved intermediates accumulate when accessibility is impaired, promoting aberrant histone modifications, loss of heterochromatin marks, and global chromatin disorganization. These epigenetic alterations reinforce epigenetic drift, a progressive loss of regulatory precision that is a hallmark of aging tissues [175]. A major outcome of failed crosstalk is the release of single-stranded DNA or fragments from collapsed forks and double-strand breaks into the cytoplasm. These extranuclear DNA species are detected by the cGAS–STING pathway, which triggers type I interferon signaling and establishes SASP. This inflammatory program not only drives chronic tissue inflammation but also amplifies replication stress and accelerates stem cell exhaustion [168,176]. The result is a vicious cycle: helicase and nuclease decline promotes genomic instability; instability feeds back into epigenetic deregulation; and both together sustain chronic inflammatory signaling. Over time, this feedback loop cements a trajectory toward cellular senescence, stem cell attrition, and organismal aging, directly linking genome–epigenome disruption with loss of regenerative capacity and tissue decline [175,177].

## 7. From DNA Damage to Senescence: The Vicious Cycle of Stem Cell Exhaustion and Aging

Throughout life, cells accumulate DNA damage from endogenous metabolic byproducts such as reactive oxygen species and replication errors, as well as from exogenous sources including ultraviolet radiation and environmental stressors. In youth, robust DNA repair systems counteract much of this injury. However, the efficiency and fidelity of these pathways gradually decline with advancing age, and the ability to maintain genomic stability differs between individuals and species [178]. Stem cells are especially susceptible to these cumulative challenges because of their long-term self-renewal and differentiation capacity. When their genomic integrity is compromised, their ability to sustain tissue homeostasis diminishes, creating conditions that accelerate aging and age-related decline [179].

Stem cells are undifferentiated cells essential for development, tissue repair, and maintenance throughout life. Their most defining features are their self-renewal capacity, allowing them to perpetuate their population over time, and their plasticity, enabling them to differentiate into the diverse cell types required for an organism’s functions [180]. These cells can be broadly categorized into two main types: pluripotent stem cells (PSCs) and adult stem cells. Pluripotent stem cells such as embryonic stem cells (ESCs) and induced pluripotent stem cells (iPSCs) can generate all the cell types in the body but cannot form extra-embryonic tissues like the placenta. In contrast, adult stem cells are typically multipotent, meaning they can differentiate into a limited range of cell types, usually within a specific tissue or organ. For example, hematopoietic stem cells (HSCs) can differentiate into various blood cells, and mesenchymal stem cells (MSCs) can generate bone, cartilage, and fat cells [181].

As stem cells age, they accumulate DNA damage and undergo epigenetic changes that impair their regenerative capacity [182]. This genomic instability arises from both intrinsic and extrinsic factors such as oxidative stress and replication errors. Over time, the accumulated DNA damage activates the DDR, a protective mechanism aimed at repairing the lesions. However, when the damage is too extensive or persistent, the DDR pathways can induce cell cycle arrest or apoptosis to prevent the propagation of damaged cell [183]. As DNA damage and stress accumulate, stem cells progressively lose their capacity for self-renewal and differentiation, a decline known as stem cell exhaustion. This impairs tissue homeostasis and regeneration: in rapidly renewing tissues like blood, skin, and gut it disrupts constant turnover, while in skeletal muscle, heart, and brain it limits repair after injury. Over time, this exhaustion accelerates tissue degeneration and contributes to age-related decline [184].

**Figure 6:**
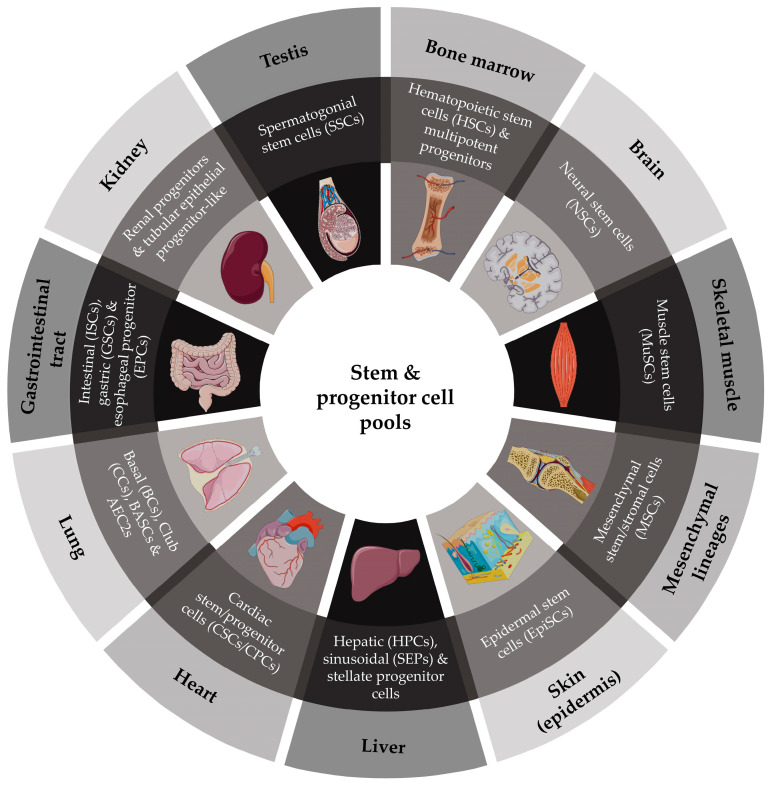
Tissue-specific stem/progenitor cell pools in human organs. Schematic representation of the principal stem and progenitor cell compartments that sustain tissue homeostasis across different organs and systems. Major pools include hematopoietic stem cells (HSCs) and multipotent progenitors in the bone marrow; neural stem cells (NSCs) in the subventricular zone and hippocampus; muscle stem cells (MuSCs, satellite cells) beneath the basal lamina of myofibers; mesenchymal stromal/stem cells (MSCs) within the bone marrow and connective tissues; epidermal stem cells (EpiSCs) in the basal epidermal layer; hepatic progenitor cells (HPCs), sinusoidal endothelial progenitors (SEPs), and stellate progenitor cells in the liver; cardiac stem/progenitor cells (CPCs) within the myocardium; pulmonary progenitors including alveolar epithelial type II cells (AEC2s), bronchoalveolar stem cells (BASCs), and basal/club cells in the airways; intestinal stem cells (ISCs), gastric stem cells (GSCs), and esophageal progenitors (EPCs) in the gastrointestinal tract; renal progenitors and tubular epithelial progenitor-like cells in the kidney; and spermatogonial stem cells (SSCs) in the testis. These stem/progenitor pools provide the regenerative backbone of tissue renewal, yet they are progressively compromised with age and in progeroid syndromes.

**Figure 7:**
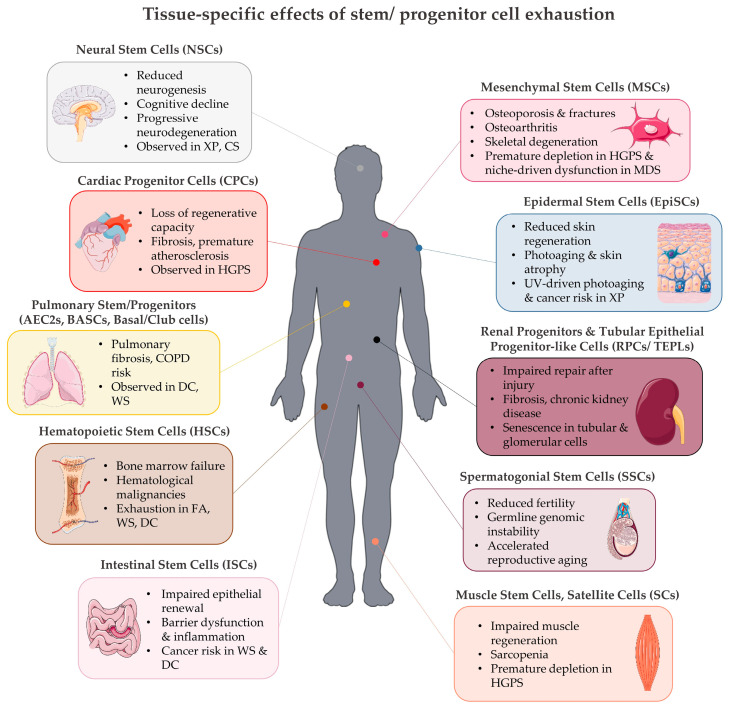
Tissue-specific effects of stem/progenitor cell exhaustion. Overview of the functional consequences of stem/progenitor cell exhaustion across organ systems. Hematopoietic stem cell (HSC) depletion leads to bone marrow failure and hematological malignancies, as observed in Fanconi anemia (FA), Werner syndrome (WS), and Dyskeratosis congenita (DC). Neural stem cell (NSC) exhaustion causes reduced neurogenesis, cognitive decline, and progressive neurodegeneration, as seen in Xeroderma Pigmentosum (XP) and Cockayne syndrome (CS). Mesenchymal stem cell (MSC) depletion drives osteoporosis, fractures, osteoarthritis, and skeletal degeneration, with premature loss in Hutchinson–Gilford progeria syndrome (HGPS) and niche dysfunction in myelodysplastic syndromes (MDS). Epidermal stem cell (EpiSC) dysfunction impairs skin regeneration, accelerates photoaging, and increases cancer susceptibility in XP. Muscle stem cell (MuSC) exhaustion promotes sarcopenia and impaired muscle regeneration, with accelerated depletion in HGPS. Spermatogonial stem cell (SSC) decline reduces fertility, induces germline genomic instability, and drives reproductive aging. Intestinal stem cell (ISC) impairment leads to epithelial dysfunction, inflammation, and cancer risk in WS and DC. Pulmonary stem/progenitor cell exhaustion, including AEC2s, BASCs, and basal/club cells, causes pulmonary fibrosis and COPD risk in DC, CS, and WS. Renal progenitors and tubular epithelial progenitor-like cells exhibit impaired repair, fibrosis, and chronic kidney disease due to senescence in tubular and glomerular compartments. Cardiac progenitor cell (CPC) decline reduces regenerative capacity, drives fibrosis and premature atherosclerosis, with accelerated dysfunction in HGPS. Collectively, these examples highlight stem/progenitor cell exhaustion as a unifying mechanism of tissue degeneration in both physiological aging and DNA repair-deficient progeroid syndromes.

Among the most intensively studied examples are hematopoietic stem cells (HSCs), the bone marrow pool responsible for generating all blood and immune lineages (Figure 6). This compartment is hierarchically organized: long-term HSCs (LT-HSCs) provide lifelong self-renewal, while short-term HSCs (ST-HSCs) and multipotent progenitors give rise to differentiated lineages with more limited capacity. With advancing age, HSCs show a progressive decline in self-renewal and differentiation potential as DNA damage accumulates over time [185]. Fanconi anemia (FA), a genetic disorder known for its DNA repair deficiencies, exemplifies the detrimental effects of impaired HSC function. In FA, mutations in nucleases like ERCC1-XPF and FANCQ hinder the repair of interstrand crosslinks (ICLs), a critical process for maintaining DNA integrity [52]. This impairment accelerates hematopoietic stem cell (HSC) depletion, leading to increased susceptibility to bone marrow failure and hematological malignancies. Evidence suggests that, beyond defective DNA repair, additional mechanisms such as oxidative stress, aldehyde accumulation, and impaired niche interactions exacerbate genomic instability and accelerate HSC exhaustion [186]. A similar phenotype is observed in Werner syndrome (WS), where mutations in the WRN helicase drive premature HSC depletion and cellular senescence. In WS, this exhaustion is amplified by oxidative injury, mitochondrial dysfunction, and replicative stress, which together promote myelodysplastic transformation and reduce lifespan [187]. Likewise, in Dyskeratosis Congenita (DC) progeria syndrome, mutations in genes related to telomere maintenance, such as *TERC* and *TERT*, impair DNA repair and lead to telomere shortening, causing HSC exhaustion and bone marrow failure [37] (Figure 7).

Neural stem cells (NSCs), residing in specialized niches such as the subventricular zone (SVZ) of the lateral ventricles and the hippocampal dentate gyrus, constitute the main regenerative pools of the brain (Figure 6). With age, these NSCs accumulate DNA damage that impairs their self-renewal and differentiation, leading to reduced neurogenesis and cognitive decline [188]. The impact of defective DNA repair on NSC maintenance is particularly evident in progeroid syndromes, which serve as paradigms of accelerated aging. In Xeroderma Pigmentosum (XP), mutations in genes encoding nucleotide excision repair nucleases (e.g., *XPG*/*ERCC5*) or helicases (*XPB*/*ERCC3*, *XPD*/*ERCC2*) prevent removal of bulky DNA lesions, leading to persistent genomic instability. This not only drives skin cancer susceptibility but also contributes to progressive neurodegeneration, with approximately one quarter of patients developing cognitive decline and sensorineural deficits [44]. A related paradigm is Cockayne syndrome (CS), caused by mutations in *CSA* or *CSB*, the latter encoding a DNA helicase. Defective transcription-coupled repair in CS leads to widespread neuronal loss, cerebellar atrophy, and demyelination, culminating in cognitive impairment and systemic premature aging. Unlike XP, CS patients are not predisposed to cancer, but instead illustrate how defective DNA repair pathways drive neurodegeneration and cachexia as hallmarks of accelerated aging [59] (Figure 7).

Skeletal muscle regeneration depends on muscle stem cells (MuSCs), also known as satellite cells, which reside beneath the basal lamina of myofibers (Figure 6). These cells are indispensable for muscle repair following injury, yet their regenerative potential declines markedly with age. Over time, MuSCs accumulate DNA damage, particularly from oxidative stress generated during muscle contraction and repeated repair cycles. In aging models, this leads to a diminished ability to proliferate and differentiate into new myocytes, thereby contributing to age-related muscle atrophy, or sarcopenia [189]. Consequently, aged MuSCs display impaired regenerative responses after injury, as shown most clearly in mouse models, and this dysfunction is thought to contribute to the progressive muscle weakness and frailty observed in elderly humans [65]. A striking example is Hutchinson-Gilford Progeria Syndrome (HGPS), where a mutation in the *LMNA* gene accelerates aging and drives premature stem cell exhaustion, including MuSC depletion, which results in early-onset muscle degeneration [65]. Beyond DNA damage, aged MuSCs also show a tendency to adopt alternative differentiation fates, such as adipogenic or fibrogenic lineages. This shift promotes fibrosis and fat deposition within muscle tissue, further compounding the progressive loss of muscle mass and function [190] (Figure 7).

Bone, cartilage, and adipose tissues are maintained by mesenchymal stem/stromal cells (MSCs) in the bone marrow and connective niches (Figure 6). With age, MSCs show reduced proliferative fitness and a skewed differentiation potential. In particular, bone marrow MSCs increasingly favor adipogenic over osteogenic lineages, leading to impaired bone formation, loss of skeletal integrity, and heightened fracture risk, as seen in osteoporosis and osteoarthritis [191]. This decline is not confined to physiological aging but also arises prematurely in genetic disorders. In Hutchinson-Gilford Progeria Syndrome (HGPS), for example, MSCs undergo premature depletion, displaying reduced proliferative capacity and impaired differentiation that exacerbate early skeletal degeneration and other hallmark tissue abnormalities [192]. Likewise, in Myelodysplastic Syndromes (MDS), pathological changes in the bone marrow niche drive premature MSC exhaustion, marked by impaired osteogenic differentiation, slow growth, and limited passage capacity. MDS represents a group of clonal bone marrow disorders characterized by ineffective hematopoiesis, genomic instability, and progressive marrow failure, providing a pathological model of premature tissue aging. Together, these findings underscore how MSC exhaustion accelerates tissue degeneration in both normal aging and disease contexts [193] (Figure 7).

Epidermal stem cells (EpiSCs), located in the basal layer of the epidermis, form the pool responsible for lifelong skin renewal and barrier maintenance (Figure 6). Unlike many other adult stem cell populations, EpiSCs display a remarkable resistance to chronological aging, retaining proliferative capacity and telomere stability even in advanced age [194]. However, their function is particularly vulnerable to environmental factors, especially ultraviolet radiation (UVR), which induces direct DNA damage, as well as oxidative lesions through reactive oxygen species. Persistent UV exposure impairs DNA repair pathways, disrupts stem cell niches, and accelerates epidermal stem cell depletion, ultimately leading to photoaging, skin atrophy, and increased cancer susceptibility [195]. A striking example is Xeroderma Pigmentosum (XP), a disorder caused by mutations in nucleotide excision repair genes. These include helicases such as XPD (ERCC2) and XPB (ERCC3), which unwind DNA during repair, and nucleases such as XPF (ERCC4) and XPG (ERCC5), which cleave damaged DNA strands. Defects in these enzymes render epidermal stem cells unable to repair UV-induced lesions, leading to premature skin aging, severe photosensitivity, and a dramatically increased risk of skin cancers [45]. Thus, while EpiSCs may resist intrinsic aging mechanisms, their long-term functionality is compromised by extrinsic genotoxic stress, making them central players in skin aging (Figure 7).

Liver regeneration is supported primarily by hepatic progenitor cells (HPCs), located in the canals of Hering, which can differentiate into both hepatocytes and cholangiocytes. Additional progenitor-like populations, including sinusoidal endothelial progenitors (SEPs) that give rise to liver sinusoidal endothelial cells (LSECs) and hepatic stellate cells (HSCs), complement this pool by sustaining vascular integrity, extracellular matrix remodeling, and the regenerative niche (Figure 6). Together, these populations underlie the liver’s extraordinary capacity for renewal. However, with advancing age, their proliferative activity, activation, and differentiation decline markedly, impairing tissue repair and predisposing to fibrosis, cirrhosis, and metabolic dysfunction [196]. Recent in vivo findings further clarify the molecular basis of this regenerative decline, showing that aging hepatocytes experience an ATR-dependent reduction in replication-origin firing during regeneration, which limits their ability to re-enter the cell cycle and sustain tissue repair [197]. As a result, impaired HPC activity limits hepatocyte and bile duct renewal, while exhausted endothelial progenitors lead to vascular rarefaction and sinusoidal stiffness. At the same time, senescent stellate cells adopt a profibrotic phenotype, which in turn accelerates fibrosis and cirrhosis. Moreover, hepatocytes themselves accumulate telomere shortening, mitochondrial dysfunction, and persistent DNA damage. These alterations further destabilize repair fidelity and ultimately drive cellular senescence [198]. Consequently, liver regeneration falters, metabolism becomes dysregulated, and the risk of chronic pathologies such as steatosis, cirrhosis, and hepatocellular carcinoma rises sharply. Progeroid syndromes offer telling examples of this process. For instance, Werner syndrome is often accompanied by non-alcoholic fatty liver disease and steatohepatitis with early fibrosis, reflecting metabolic dysfunction and impaired repair [199]. Likewise, mouse models of Hutchinson–Gilford progeria show premature hepatic steatosis and fibrosis, reflecting how nuclear and DNA repair defects compromise liver regenerative capacity; similar, though less consistently reported, findings have been described in human HGPS patients [200] (Figure 7).

A comparable pattern is observed in the heart, which, despite once being considered a post-mitotic organ, is now recognized to harbor resident progenitor-like cardiac cell populations (CSCs/CPCs) with limited regenerative potential [201] (Figure 6). These include c-kit^+^ clonogenic CSCs, cardiosphere-derived cells (CDCs), and other progenitor populations that together sustain limited cardiomyocyte turnover and contribute to vascular regeneration throughout life [202]. Functionally, these cells display multipotent capacity, giving rise to cardiomyocytes, smooth muscle cells, and endothelial cells [203]. With advancing age, however, CPCs accumulate hallmarks of cellular senescence, including telomere attrition, mitochondrial dysfunction, and persistent DNA damage, which progressively impair their proliferative and differentiation potential [204]. As these pools decline, cardiac regenerative capacity is lost, and CPC exhaustion contributes to myocardial fibrosis, reduced vascularization, and progression toward heart failure [203]. Progeroid syndromes reinforce this link: histological analyses of patients with Hutchinson–Gilford progeria syndrome (HGPS) reveal an accelerated form of cardiovascular aging, marked by extensive adventitial fibrosis, premature atherosclerosis, and structural myocardial alterations [205] (Figure 7).

The lung provides another important case of age-related stem cell exhaustion. Pulmonary renewal depends on distinct region-specific stem/progenitor pools: basal cells and club cells in the airways, bronchoalveolar stem cells (BASCs) at the bronchioalveolar duct junction, and alveolar type II epithelial cells (AEC2s) within the alveoli. Among these, AEC2s most closely resemble a true stem cell population, as they not only produce surfactant but also give rise to alveolar type I cells that form the gas-exchange surface [206] (Figure 6). With advancing age, these populations undergo telomere shortening, accumulate DNA damage, and develop mitochondrial dysfunction. As a result, their renewal capacity declines and their ability to restore alveolar structure after injury is impaired [207]. AEC2 senescence in particular has been implicated in chronic lung diseases such as idiopathic pulmonary fibrosis (IPF) and COPD, where progenitor exhaustion and maladaptive repair drive progressive tissue remodeling and loss of function. Progeroid syndromes provide further examples: in Dyskeratosis Congenita, mutations in telomerase or shelterin components cause telomere attrition in alveolar progenitors, leading to pulmonary fibrosis [208] and in Werner syndrome, loss of the WRN helicase accelerates interstitial lung disease and fibrosis, underscoring how DNA repair failure destabilizes lung homeostasis [209] (Figure 7).

The gastrointestinal epithelium is maintained by distinct stem and progenitor pools that sustain continuous renewal along the digestive tract. The best-defined are intestinal stem cells (ISCs) located at the crypt base, which drive constant replenishment of the small intestine and colon lining. Complementing them are gastric stem cells (GSCs) in the stomach glands, responsible for epithelial turnover in the gastric mucosa, and esophageal progenitor cells (EPCs) in the basal layer of the esophageal epithelium, which preserve squamous integrity [210,211] (Figure 6). With aging, these populations display reduced proliferative capacity, impaired differentiation, and disrupted niche signaling, processes linked to replication stress, mitochondrial dysfunction, and DNA damage accumulation [212]. As a result, epithelial renewal slows, barrier function deteriorates, and susceptibility to inflammation and tumorigenesis rises. Progeroid syndromes provide further evidence: patients with Werner syndrome (WS) show increased gastrointestinal tumor risk and mucosal atrophy, while those with Dyskeratosis congenita (DC), a telomeropathy, frequently develop esophageal stenosis and gastrointestinal cancers due to premature telomere attrition and stem cell depletion [213,214] (Figure 7).

Renal homeostasis relies on tubular epithelial cells with progenitor-like properties and small pools of resident renal progenitors that participate in repair after injury (Figure 6). Although epithelial turnover under baseline conditions is limited, these cells become essential following insults such as ischemia–reperfusion or nephrotoxic damage. With aging, their proliferative reserve declines, apoptosis increases, and maladaptive repair responses emerge, fostering fibrosis and predisposing to chronic kidney disease progression [215]. Cellular senescence, driven by telomere shortening, oxidative stress, and activation of p16INK4a /p21 checkpoints, accumulates in tubular and glomerular compartments, restricting regenerative potential and diminishing functional recovery after injury [216] (Figure 7). In parallel, reproductive aging highlights a similar dynamic. Spermatogonial stem cells (SSCs), located along the basement membrane of seminiferous tubules, sustain spermatogenesis by balancing self-renewal with differentiation (Figure 6). With advancing age, SSCs show reduced proliferation, telomere attrition, and increasing DNA damage, while extrinsic alterations in the testicular niche further impair their function. This exhaustion reduces sperm output, increases genomic instability in the germline, and shortens reproductive longevity [217] (Figure 7).

All these examples of stem cell exhaustion across various tissues highlight a common underlying process: the accumulation of DNA damage and epigenetic alterations ultimately drive the cells into a state of senescence. Senescence represents an irreversible cell cycle arrest, activated when damage becomes irreparable, and functions as a tumor-suppressive mechanism to prevent the propagation of genetically unstable cells [4]. However, while this response protects against malignant transformation, it simultaneously contributes to organismal aging. Senescent cells display a distinct phenotype, characterized not only by growth arrest and resistance to apoptosis but also by profound metabolic and transcriptional changes that reshape their function within tissues [218].

Mechanistically, cellular senescence can be triggered by various forms of stress, including telomere shortening, oxidative damage, oncogenic activation, and persistent DNA damage signaling. At the molecular level, two major pathways orchestrate this process: the p53/p21 and p16INK4a /Rb axes. In the first, genotoxic or metabolic stress activates p53, which induces the expression of p21, a cyclin-dependent kinase inhibitor that halts the cell cycle by blocking cyclin–CDK complexes at both the G1/S and G2/M checkpoints [219]. This early response allows the cell to pause and attempt repair; if the damage cannot be resolved, sustained p21 activity locks the cell into a senescent state. The p16INK4a/Rb pathway reinforces this arrest by preventing CDK4/6–cyclin D complex formation and maintaining the retinoblastoma protein in its inactive form [220]. While p21 is typically associated with an early and transient phase of senescence, p16INK4a marks a later and more stable stage, reflecting the dynamic and heterogeneous nature of senescent cell populations [219].

Senescent cells remain metabolically active and develop a senescence-associated secretory phenotype (SASP), that alters intercellular communication and tissue homeostasis. The SASP is driven by signaling pathways such as NF-κB, p38 MAPK, mTOR, and cGAS–STING, which connect persistent DNA-damage signaling to inflammatory and remodeling responses [221]. Initially, the SASP aids tissue repair and immune surveillance, but its chronic persistence drives a pro-inflammatory milieu that promotes fibrosis, matrix degradation, and functional decline [222]. This creates a vicious cycle: senescent cells impair tissue homeostasis, which reduces regenerative potential and further accelerates the accumulation of senescent cells. Over time, this loop contributes to degenerative conditions such as osteoarthritis, sarcopenia, neurodegeneration, and cardiovascular disease [220,223].

Recent evidence further reveals that p21-dependent senescence (PASP) differs qualitatively from the p16INK4a -associated phenotype, not only in timing but also in secretory profile and physiological outcome. Early p21 activation induces transient secretion of chemokines, facilitating macrophage-mediated clearance of damaged cells and promoting regeneration. In contrast, sustained p21 expression or delayed transition to p16INK4a -driven senescence results in persistent SASP activity and progressive tissue decline [219]. These mechanistic insights align with the broader concept that senescence is not a uniform endpoint but a dynamic and context-dependent process, whose outcome depends on the duration and intensity of DDR signaling.

Senescence therefore represents a biological paradox: it acts as a safeguard against malignant transformation and supports normal development, yet its persistence over time contributes to tissue dysfunction and accelerates the aging process [221]. The growing understanding of how stem cell exhaustion, p21/p16 signaling and SASP heterogeneity interact to promote degeneration has opened new perspectives for therapeutic intervention. Rather than viewing senescence solely as detrimental, current research frames it as a process that, if properly modulated, could help preserve tissue homeostasis and delay the onset of age-related decline [218]. This recognition has shifted attention toward the development of therapeutic approaches aimed at mitigating the negative impact of senescence and restoring balance in aging tissues. Such strategies provide the conceptual bridge to broader pharmacological interventions against aging, which encompass diverse mechanisms and target different aspects of the DDR–aging axis.

**Figure 8:**
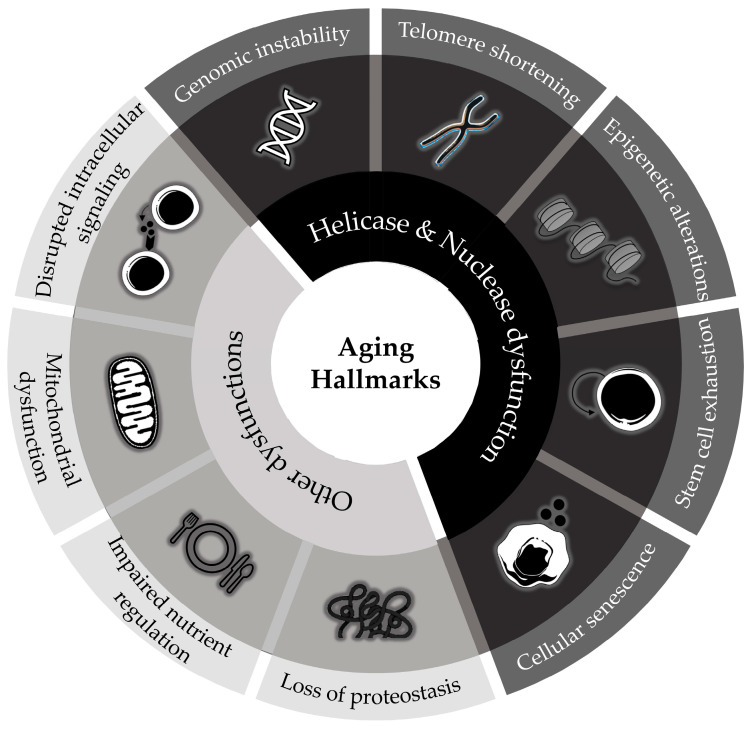
Interconnection between helicase and nuclease dysfunction and the hallmarks of aging. Schematic representation illustrates how impaired helicase and nuclease activity converges on the principal hallmarks of aging. Defects in these enzymes compromise genomic stability and telomere maintenance, induce replication stress, and promote downstream alterations such as epigenetic dysregulation, cellular senescence, and stem-cell exhaustion.

Collectively, the evidence discussed throughout this chapter underscores that disturbances in the DNA damage response are central to the aging process. In particular, dysfunctions of helicases and nucleases exemplify how impaired genome maintenance translates into the biological hallmarks of aging. Their loss of function amplifies replication stress and genomic instability, driving cellular senescence, stem-cell exhaustion, and systemic decline. To integrate these mechanistic insights, Figure 8 summarizes the impact of helicase and nuclease dysfunction across the principal hallmarks of aging, ultimately linking molecular damage to organismal deterioration. Understanding these interconnected pathways establishes the rationale for the development of pharmacological interventions that target the DDR–aging network.

## 8. Therapeutic Strategies Targeting the DDR–Aging Axis

Since ancient times, research has been devoted to the identification of anti-aging drugs. Anti-aging drugs are pharmacological interventions designed to delay age-related decline and extend healthspan [224]. As previously stated, aging is a complex process with various mechanisms implicated in its progression. Because these mechanisms vary in intensity across tissues and individuals, age-related diseases display significant variability, requiring the design of a variety of therapeutic strategies tailored to the specific impairment mechanism. The pharmacological interventions targeting aging and age-related diseases can be summarized into five major categories including antioxidants, anti-inflammatory drugs, metabolic and proteostatic modulators, genome and epigenome stability modulators and senescence-targeting therapies [225].

Among these categories, only some directly modulate the DDR, which is central to genome instability and senescence. The therapeutic rationale of these interventions is derived from the observation that unrepaired DNA damage activates checkpoint pathways, leading to cell cycle arrest, senescence, and ultimately tissue dysfunction [226]. The pharmacological strategies targeting the DDR–aging axis can be grouped according to whether they act directly on DDR pathways, downstream on DDR-induced senescence or indirectly by reducing the upstream burden of DNA damage.

The most direct therapeutic strategies involve the modulation of DNA damage response pathway checkpoints. It has been demonstrated that while DDR pathways are tumor suppressive in youth, their chronic activation leads to stem cell exhaustion and tissue dysfunction with age. Experimental studies in mouse models with telomere dysfunction have shown that targeting checkpoint proteins (e.g., p21, PUMA, EXO1, p16INK4a) can extend lifespan and improve tissue maintenance, making DDR checkpoints both a mechanistic driver of aging and a therapeutic target for age-related diseases [226]. ATM/ATR are at the upstream level of these responses and selective inhibitors have been developed, but with oncology indications, and their potential anti-aging effects remain under investigation [227]. Recent in vivo evidence in aged mouse liver has shown that pharmacologic ATR inhibition can restore replication-origin firing suppressed by the aging process, indicating that checkpoint hyper-activation directly limits DNA replication initiation in old tissues. However, the same intervention failed to reinstate youthful levels of hepatocyte proliferation and instead provoked inflammatory responses, underscoring that systemic ATR inhibition may relieve replication stress at the expense of tissue homeostasis [197]. In addition, restoration of NAD^+^ levels, a critical metabolic co-enzyme that reduces with age, through supplementation with precursors such as nicotinamide riboside (NR) and nicotinamide mononucleotide (NMN), has emerged as one of the most promising pharmacological strategies to counteract age-related decline. These interventions sustain the activity of PARPs and sirtuins by replenishing NAD^+^ pools, thereby supporting DNA repair, mitochondrial function, and cellular stress resistance [228]. Direct sirtuin activation is also a potent therapeutic strategy. In preclinical and early clinical studies, activators like resveratrol, SRT1720, and SRT2104 have shown improvements in metabolic and inflammatory markers. However, the consistent lifespan extension that these activators promote remains unproven [229]. Finally, epigenetic modulation also reinforces DDR fidelity. Aging involves shifts in DNA methylation, histone marks, and heterochromatin loss that impair repair efficiency. Studies using Histone Deacetylase (HDAC) inhibitors (vorinostat, panobinostat), DNA Methyltransferase (DNMT) inhibitors (azacitidine, decitabine), and experimental Bromodomain and extra-terminal (BET) inhibitors (JQ1), demonstrate the possibility of pharmacologically reshaping the epigenome, though toxicity remains a barrier [230]. Together, these targets illustrate how modulation of the DDR checkpoint signaling and repair capacity provides a mechanistically grounded framework for pharmacological intervention in aging.

A second group of strategies focuses on downstream DDR-induced senescence. Senescent cells are characterized by irreversible cell-cycle arrest, which is predominantly driven by p21 and/or p16INK4a, morphological alterations, resistance to apoptosis, modified gene signaling pathways, and the formation of SASP [231]. These features have made senescent cells and their secretome major therapeutic targets in aging. One approach is the use of senolytics, which selectively eliminate senescent cells by targeting their anti-apoptotic pathways. The first reported senolytic regimen was a combination of dasatinib and quercetin, which has been shown to alleviate physical dysfunction and extend the lifespan of aged mice [232]. Other senolytics include navitoclax (ABT-263), an inhibitor that targets anti-apoptotic members of the BCL-2 family, particularly BCL-2, BCL-W that restores the apoptosis of senescent cells, though its clinical use is limited by dose-dependent thrombocytopenia [233]. Also, worth mentioning is the flavonoid fisetin that acts as a natural senolytic, reducing senescent cell burden and SASP factors in mice, extending lifespan, and also decreasing senescent cells in human adipose explants, with several clinical trials now underway [234]. Complementary to senolytics are senomorphics, agents that aim to modulate senescence cells’ phenotype and secretome, without inducing cell death [235]. Among senomorphics, rapamycin is the best-established agent, suppressing SASP through mTORC1 inhibition and extending lifespan in multiple organisms [236]. Metformin likewise reduces senescence and SASP via AMPK-dependent and NF-κB-modulating pathways, extending lifespan in model organisms. Also, epidemiological data suggest reduced mortality and age-related disease incidence in humans, making it the most clinically advanced drug [237]. Other senomorphic agents, such as ruxolitinib, a JAK1/2 inhibitor, further highlight the potential of targeting SASP signaling, as it has improved bone strength, reduced inflammation and delayed premature aging phenotypes in progeria models [238]. Additional experimental senomorphics, including NF-κB and p38 MAPK inhibitors, represent emerging targets for SASP suppression in preclinical models [233]. Beyond direct modulation of senescent cells, stem cell-based regenerative therapies represent another strategy to counteract the downstream effects of chronic DDR activation. Transplantation of mesenchymal or hematopoietic stem cells, which retain the capacity for self-renewal and multilineage differentiation, has been demonstrated to restore lost regenerative capacity in cases of chronic DDR activation [239]. In addition, ex vivo partial reprogramming and interventions targeting the aged stem cell niche have also been shown to rejuvenate aged cells and improve tissue repair in animal models [240]. Collectively, these senotherapeutic and regenerative strategies illustrate how targeting DDR-induced senescence and its consequences can alleviate tissue dysfunction and potentially extend healthspan.

Finally, several interventions indirectly affect the DDR by reducing cellular damage or enhancing tolerance to genotoxic stress. Antioxidant interventions, such as N-acetylcysteine (NAC) and mitochondria-targeted compounds (MitoQ, CoQ10, SkQ1), have been shown to mitigate oxidative stress, reduce DNA damage, and delay the onset of cellular senescence in preclinical models [241]. Complementing these pharmacological approaches, naturally occurring dietary antioxidants contribute to genomic stability and defense against oxidative stress during aging. Vitamins (A, C, D, E, K, and B-complex), selenium, and zinc support redox balance, telomere integrity, and DNA repair enzyme activity [242]. Similarly, nutritional and metabolic interventions, including caloric restriction and fasting-mimicking diets, have been demonstrated to decrease metabolic ROS production and enhance genome maintenance, resulting in extending healthspan and lifespan across diverse organisms [243]. Also, polyphenol-rich diets, exemplified by the Mediterranean pattern, provide compounds like resveratrol, quercetin, and curcumin that enhance DNA repair and reinforce cellular stress resilience [244]. Another indirect approach is targeting chronic inflammation. Blocking IL-1 or IL-6, which is already clinically feasible with anakinra and tocilizumab, respectively, restores hematopoietic regeneration and improves tissue homeostasis in aging models [245]. Finally, the targeting of NRF2, a regulator of redox balance, inflammation, and genome stability, offers another therapeutic avenue. Pharmacological activators including sulforaphane, dimethyl fumarate, and bardoxolone methyl restore NRF2 activity, thereby enhancing cellular defences against genotoxic stress and delaying senescence [246]. Collectively, these indirect strategies do not directly modulate DDR signaling but instead reduce upstream sources of DNA damage or improve stress resilience, thereby complementing direct DDR-targeting and senescence-focused interventions. These therapeutic strategies, their representative agents, molecular targets, and principal effects, are summarized in Table 3.

Taken everything into account, the therapeutic strategies targeting the DDR–aging axis encompass a range of approaches, including direct checkpoint and repair modulation, downstream senescence-targeting methods, and indirect interventions that aim to reduce DNA damage and enhance genomic instability resistance. This framework highlights that genome maintenance can be promoted at multiple biological levels. Although numerous agents, including rapamycin, metformin, NAD^+^ precursors, and senolytics, have demonstrated consistent benefits in preclinical models, their translation into clinically safe and effective anti-aging therapies remains a central challenge [247]. Issues of specificity, off-target effects, toxicity, and long-term safety demand careful refinement, in parallel with the development of reliable biomarkers to monitor biological aging and therapeutic response [248]. In addition to these pharmacological strategies, naturally derived products offer complementary, low-toxicity approaches that reinforce redox balance, enhance DNA repair capacity, and strengthen cellular defence against age-related stressors. Nonetheless, the combination of these interventions highlights DDR modulation as a central therapeutic principle in anti-aging research, offering a mechanistically grounded and increasingly translational path to extending healthspan and delaying age-related disease.

## 9. Discussion

The collective evidence from genetic, molecular, and experimental studies demonstrates that genome instability is not a secondary outcome but a fundamental driver of aging. Helicases and nucleases form the enzymatic core of genome maintenance, coordinating DNA replication, repair, and chromatin organization to preserve the balance between stability and adaptability required for tissue renewal. When this coordination declines, transient DNA damage becomes chronic, sustaining DDR activation, cellular senescence, and stem cell exhaustion. The molecular disruptions observed in progeroid syndromes are analogous to the gradual deterioration that accompanies physiological aging, suggesting that both inherited and natural aging may have a unified genomic basis. The progressive loss of helicase–nuclease function thus represents a convergent mechanism linking DNA instability, telomere erosion, and the decline of regenerative capacity.

This convergence provides a foundation for the development of intervention strategies. Emerging strategies aim to modulate the DNA damage response and reinforce genome stability in a controlled manner. Strategies aimed at reducing chronic cellular stress, enhancing repair fidelity, and maintaining chromatin and metabolic balance have the potential to delay functional decline and preserve stem cell competence. The challenge lies in strengthening genome maintenance without disturbing tumor-suppressive barriers or promoting unchecked proliferation. Achieving this balance will require integrating structural, genomic, and physiological insights to map how repair systems adapt across tissues and time. Ultimately, understanding how these enzymatic networks uphold genome integrity provides both a molecular explanation for aging and a foundation for interventions that extend healthspan by stabilizing the very processes that sustain homeostasis.

## Figures and Tables

**Table 1:** Major human progeroid syndromes classified according to the underlying DDR defect. Mutation nomenclature follows HGVS conventions (“c.” = coding DNA, “n.” = non-coding DNA sequence or transcript, “p.” = protein, “*” = stop codon).

Category	Syndrome	Gene/Protein	Frequent Mutations	Molecular Functions	Aging Phenotypes/Senescence Link	Ref.
**Defects in Helicase Function**	**Werner Syndrome (WS)**	*WRN* (RecQ Helicase & Exonuclease)	c.1105C>T (p.Arg369*, nonsense, exon 9) & c.3139-1G>C (splice-site, exon 26) >70 *WRN* variants	Resolves complex DNA structures (e.g., G-quadruplexes); telomere maintenance; DSB repair regulation (c-NHEJ vs. alt-NHEJ)	Hair graying, cataracts, osteoporosis, atherosclerosis, cancer; linked to telomere shortening and persistent DDR, drivers of senescence	[20,21,22,23,24]
	**Bloom Syndrome (BS)**	*BLM* (RecQ Helicase)	c.2207_2212del6ins7 (BLMAsh, exon 10) & c.1642C>T (p.Gln548*, nonsense, exon 7), >150 *BLM* variants	Suppresses aberrant homologous recombination; dissolves double Holliday junctions via the BTR complex; ensures replication fork stability and genome maintenance under replication stress	Does not present classical premature aging traits, but is characterized by short stature, proportional growth deficiency, immunodeficiency, sun-sensitive skin changes, and very high cancer predisposition.	[20,25,26,27,28,29]
	**Rothmund Thomson Syndrome (RTS)**	*RECQL4* (RecQ Helicase)	c.1573delT (p.Cys525Alafs*33, frameshift, exon 9) & c.2269C>T (p.Arg757*, nonsense, exon 14), >100 *RECQL4* variants	DNA replication; DNA repair (DSB repair, HR, NHEJ); Mitochondrial DNA integrity	Poikiloderma, skeletal abnormalities, juvenile cataracts & cancer predisposition. *RECQL4* deficiency leads to DNA damage accumulation, with cellular senescence observed in vitro, although cancer predisposition is the dominant clinical outcome.	[30,31,32,33,34]
	**Dyskeratosis Congenita (DC)**	*RTEL1* (DNA helicase)	c.3791G>A (p.Arg1264His, exon 34), >70 *RTEL1* variants	Telomerase biogenesis; telomere replication and protection.	Abnormal skin pigmentation, nail dystrophy, leucoplakia, bone marrow failure, pulmonary fibrosis & liver disease. Critically short telomeres cause premature stem cell senescence, leading to progressive tissue failure.	[35,36,37]
		*TERC* (RNA component)	n.64C>G (structural RNA variant) & n.110A>G (structural RNA variant), >30 *TERC* variants			
		*TERT* (reverse transcriptase)	c.2594G>A (p.Arg865His, exon 11), >100 *TERT* variants			
		*DKC1* (RNA pseudouridine synthase)	c.1058C>T (p.Ala353Val, exon 12) >60 *DKC1* variants			
		*TINF2* (shelterin protein)	c.844C>T (p.Arg282His, exon 6), >40 *TINF2* variants			
	**Trichothiodystrophy (TTD)**	*ERCC2*/*XPD* (DNA helicase, TFIIH complex)	c.2164C>T (p.Arg722Trp, missense) & c.335G>A (p.Arg112His, exon 5, missense), >40 *ERCC2* variants	TFIIH complex component; global genome NER; transcription-coupled NER; transcription initiation.	Brittle hair and nails, progressive sensorineural deafness, photosensitivity, intellectual disability and reduced fertility. Some forms lack cancer predisposition. Defects in both NER and transcription contribute to early cellular aging.	[38,39,40,41,42]
		*ERCC3/XPB* (DNA helicase, TFIIH complex)	c.355A>C (p.Thr119Pro, exon 3), <10 *ERCC3* variants			
		*GTF2H5* (structural TFIIH subunit)	c.166C>T (p.Arg56Ter, exon 2) & c.2T>C (p.Met1Thr, start codon), <10 *GTF2H5* variants			
**Defects in Helicase & Nuclease Function**	**Xeroderma ** **Pigmentosum (XP)**	*XPA* (scaffold protein)	c.390-1G>C (splice-site, intron 3), >40 *XPA* variants	Global genome NER (bulky adduct/UV lesion removal); transcription-coupled NER (in some subtypes).	Extreme UV sensitivity with early-onset skin cancers; neurodegeneration occurs in some subtypes. Persistent unrepaired DNA damage leads to chronic DDR activation and premature cellular senescence	[43,44,45,46,47]
		*XPC* (damage recognition)	c.1643_1644delTG (p.Val548Alafs*25, exon 9), >120 *XPC* variants			
		*ERCC2/XPD* (DNA helicase, TFIIH complex)	c.1993A>G (p.Lys618Glu, exon 21) & c.2047C>T (p.Arg683Trp, exon 22), >100 *ERCC2* variants			
		*ERCC3/XPB* (DNA helicase, TFIIH complex)	Rare, <20 *ERCC3* variants			
		*ERCC4/XPF* (structure-specific endonuclease)	c.2395C>T (p.Arg799Trp, exon 8), <30 *ERCC4* variants			
		*ERCC5/XPG* (structure-specific endonuclease)	c.4G>T (p.Gly2Trp) & c.1947T>A (p.Tyr649*), <20 *ERCC5* variants			
		*POLH* (DNA polymerase eta)	c.907C>T (p.Arg304Trp, exon 8), >50 *POLH* variants			
**Other DDR defects tegoryility. Some forms lackoutvery cancer**	**Nijmegen Breakage Syndrome (NBS)**	*NBN* (scaffold protein in MRN complex)	c.657_661del5 (p.K219fsX19, exon 6), >11 *NBN* variants	MRN complex component; DSB sensing; telomere stability.	Microcephaly, immunodeficiency, cellular & humoral immunodeficiency, cancer predisposition. Genomic instability accelerates senescence, acting as a tumor-suppressive barrier.	[48,49,27]
	**Fanconi Anemia (FA)**	*FANCA*	c.3788_3790delTCT (p.Phe1263del, exon 38), > 250 *FANCA* variants	DNA interstrand crosslink (ICL) repair; replication stress protection; chromosome stability.	Progressive bone marrow failure, developmental anomalies, skin pigmentation changes & cancer predisposition. Persistent DNA damage and replication stress induces senescence, driving hematopoietic stem cell exhaustion.	[50,51,52,53,54,55]
		*FANCC*	c.456+4A>T (splice-site, intron 4), >60 *FANCC* variants			
		*FANCG*	c.307+1G>C (splice donor, intron 3), >50 *FANCG* variants			
		*FANCD1 (BRCA2)* & >20 other FA genes	c.6174delT (p.Ser1982fs, exon 11), >100 *FANCD1*			
	**Ataxia** **Telangiectasia (AT)**	*ATM* (Ataxia–Telangiectasia Mutated kinase)	c.7630-2A>C (splice-site, intron 53) & c.8147T>C (p.Val2716Ala, exon 56), >1000 *ATM* variants	ATM–DSB signaling; checkpoint control; telomere maintenance; oxidative and mitochondrial homeostasis.	Progressive neurodegeneration, immunodeficiency, cancer predisposition & radiosensitivity. Persistent DDR activation with oxidative damage and mitochondrial dysfunction drives early senescence.	[27,56,57,58]
	**Cockayne Syndrome (CS)**	*ERCC6/CSB* (ATP-dependent chromatin remodeler)	c.5254_5257del (p.Thr1752fs*6, exon 23) & c.1834C>T (p.Arg612Ter, exon 9), >150 *ERCC6* variants	Transcription-coupled NER (removal of RNA Pol II-blocking lesions); transcription regulation; chromatin remodeling.	Growth failure, neurodegeneration, sensorineural hearing loss, photosensitivity, cachectic dwarfism & no cancer predisposition. Persistent transcription-blocking lesions induce senescence.	[20,47,59,60,61,62]
		*ERCC8/CSA* (WD-repeat ubiquitin ligase component) & other *ERCC* genes	Complex exon 4 rearrangement (del/inv/ins, exon 4), >80 *ERCC8* variants			
**Defects in Nuclear Envelope**	**Hutchinson Gilford Progeria Syndrome (HGPS)**	*LMNA*	c.1824C>T (p.G608G, ex11), >20 *LMNA* variants (90% of cases due to this founder mutation)	Nuclear lamina structure; chromatin organization; telomere maintenance.	Accelerated atherosclerosis, alopecia, lipodystrophy, joint stiffness, and severe premature aging driven by persistent DDR signaling from nuclear architecture defects.	[63,64,65]
	**Mandibuloacral Dysplasia (MAD)**	*LMNA*	c.1580G>A (p.Arg527His, ex9), >20 *LMNA* variants	Lamin structure; nuclear architecture	Growth retardation, skeletal abnormalities, partial lipodystrophy, progeroid features. Nuclear envelope fragility induces chronic DDR and early senescence.	[66,67,68]
		*ZMPSTE24* (zinc metalloprotease)	c.1085dupT (p.Phe361fs, ex9), >30 *ZMPSTE24* variants			
		*POLD1* (catalytic subunit of DNA polymerase δ)	c.1812_1814del (p.Ser605del, exon 15), <10 *POLD1* variants			

**Table 2:** Representative mouse models of DDR defects and their contribution to accelerated aging. Mouse genotypes follow standard nomenclature (“−/−” = homozygous knockout, “Δ” = domain or exon deletion, “mut/mut” = homozygous point-mutant allele).

Model Type	Mouse Model	Gene/Protein	Molecular Functions	Aging Phenotypes/Senescence Link	Ref.
**Defects in Helicase Function**	*Wrn*−/− (null) *Wrn*^Δ^^hel/Δhel^(helicase-dead)	*Wrn* (RecQ helicase)	DNA replication, recombination & telomere maintenance	Null: mild or no overt aging, fertile, near-normal lifespan with only subtle senescence. *Wrn*^Δhel/Δhel^: clear aging features, reduced lifespan, increased oxidative stress, metabolic abnormalities, and cancer predisposition.	[78,79,80,81]
	*Wrn*−/− *Terc*−/−	*Wrn* (RecQ helicase) & *Terc* (telomerase RNA component)	DNA replication, recombination & telomere maintenance	Hair graying, osteoporosis, cataracts, diabetes, and cancer. Accelerated telomere erosion drives persistent DNA damage signaling and widespread senescence, closely mirroring the human Werner syndrome phenotype.	[23,82]
	*Blm*−/−*(null)* *Blm*^3/3^ (hypomorphic) *Blm*^loxP/loxP^ (conditional)	*Blm* (RecQ helicase)	Homologous recombination, suppression of sister chromatin exchange (SCE), maintenance of replication fork stability	Nulls: perinatal lethality. Hypomorphic/conditional mutants: viable, genomic instability, elevated SCE, cancer predisposition, shortened lifespan, underscoring genome instability as the driver of aging phenotypes.	[83,84]
	*Recql4*−/− (null) *Recql4*^hd/hd^ (hypo-morphic) *Recql4*^loxP/loxP^ (conditional)	*Recql4* (RecQ helicase)	DNA replication initiation, NER/NHEJ, mtDNA stability	Nulls: embryonically lethal. Hypomorphic/conditional mutants: viable, growth retardation, skeletal abnormalities, genomic instability, cancer predisposition, and early senescence. *Recql4* deficiency models Rothmund–Thomson syndrome, linking replication defects and genome instability to accelerated aging.	[85,86]
**Defects in Nuclease function**	*Ercc1*−/− (null), *Ercc1*^−^/^Δ^ (hypomorphic)	*Ercc1* (ERCC1–XPF endonuclease)	NER & interstrand crosslink (ICL) repair, supports transcription-coupled repair	Nulls: early lethality. Hypomorphs: systemic progeroid features, growth retardation, multi-organ decline & shortened lifespan. Accelerated epigenetic aging, high senescent cell burden, underscoring persistent DNA lesions and chronic DDR as drivers of senescence and tissue aging.	[74,87,88]
**Defects in Helicase & Nuclease Function**	*Xpg*−/− (null), *Xpd*^TTD/TTD^ (hypomorphic)	*Ercc5/Xpg* (structure-specific endonuclease) *Ercc2/Xpd* (DNA helicase)	Both impair NER lesion excision, TFIIH-mediated transcription initiation	Cachexia, brittle hair, neurodegeneration, retinal degeneration, growth retardation, and shortened lifespan. Mimics human trichothiodystrophy. Persistent NER defects and transcription-blocking lesions drive chronic DDR and senescence.	[89,90]
**Other DDR defects**	*Atm*−/− (null)	*Atm* (ATM kinase)	DSB signaling, checkpoint control, oxidative stress response	Growth retardation, infertility, immunodeficiency, neurodegeneration, increased tumor incidence, reduced lifespan. Persistent DSB signaling drives senescence and stem cell exhaustion, contributing to premature aging.	[91,92,93]
	*Csa*−/−, *Csb*−/−	*Ercc8/Csa* (WD40-repeat protein) *Ercc6/Csb* (ATP-dependent chromatin remodeler)	Transcription-coupled NER, transcription regulation, mitochondrial function	Growth retardation, cachexia, neurological dysfunction, kyphosis, retinal degeneration, and reduced fertility. Lifespan is shortened without marked cancer predisposition. Persistent transcription-blocking lesions drive senescence.	[94,95]
	*Prkdc*−/− (SCID mouse)	*Prkdc* (DNA-PKcs kinases)	NHEJ, V(D)J recombination, DSB repair & telomere stability	Severe combined immunodeficiency (SCID), growth retardation, osteoporosis, kyphosis, shortened lifespan. Progressive stem cell exhaustion from persistent DSBs and telomere dysfunction drives chronic DDR, p53/p21-mediated senescence, and accelerated aging.	[93,96]
	*Ku80*−/−	*Xrcc5/Ku80* (DNA end-binding protein)	NHEJ, V(D)J recombination, DSB repair & telomere stability	Growth retardation, shortened lifespan, early osteoporosis, kyphosis, stem cell exhaustion, and impaired immune development. Persistent DSBs sustain DDR activation and p53-driven senescence, accelerating systemic aging	[97,98]
	*Fanca*−/−, *Fancc*−/−, *Fancg*−/− (nulls)	*Fanca, Fancc, Fancg* (FA core complex subunits)	ICL repair, replication fork protection, telomere stability	Mice are viable and do not exhibit systemic progeroid features, but show hematopoietic stem cell attrition, progressive bone marrow failure, and heightened genotoxic sensitivity. Replication stress and unrepaired DNA damage in stem cells trigger p53/p21-mediated senescence, resulting in segmental rather than systemic aging.	[99,100]
**Defects in Nuclear Envelope (Laminopathies)**	*HGPS* knock-in models (*Lmna^HG^* & *Lmna^G609G^*)	*Lmna* (Progerin/ mutant Lamin A)	Nuclear envelope stability, chromatin organization, DDR signaling	Growth retardation, bone and cardiovascular abnormalities, reduced lifespan. Progerin accumulation disrupts nuclear architecture, induces persistent DDR activation, and promotes cellular senescence resembling human HGPS.	[75,101,102]
	*Zmpste24*−/−	*Zmpste24* (metalloprotease)	Nuclear envelope integrity (maturation of prelamin A to lamin A)	Severe progeroid phenotype with growth retardation, osteoporosis, kyphosis, muscle weakness, early death. Prelamin A accumulation disrupts nuclear architecture, induces DNA damage and senescence.	[75,103,104]
	*Lmna*−/−	*Lmna* (Lamnin A & C)	Nuclear envelope structure, chromatin organization, DDR signaling	Severe muscular dystrophy, growth retardation, and early death. Phenotype reflects developmental failure rather than systemic progeria and thus differs from HGPS knock-in and *Zmpste24*−/− models that recapitulate premature aging.	[75]
**Defects in Mitochondrial Function**	*Polg*^mut/mut^ (D257A knock-in)	*Polg* (DNA polymerase γ)	mtDNA replication & proofreading	Alopecia, kyphosis, osteoporosis, anemia, cardiomyopathy, infertility, and shortened lifespan. Mitochondrial dysfunction induces ROS imbalance and triggers persistent DDR signaling, driving cellular senescence and stem cell exhaustion.	[105,106]
**Senescence Accelerated Models**	SAMP strains	Polygenic variants, derived by selective inbreeding of AKR/J mice.	Mitochondrial dysfunction, ROS regulation & tissue homeostasis	Shortened lifespan with strain-specific pathologies: SAMP1 (amyloidosis), SAMP6 (osteoporosis), SAMP8 (neurodegeneration & amyloid-β and tau pathology). ROS-driven mitochondrial dysfunction and cellular senescence promote chronic inflammation and systemic aging.	[76,107]
**Induced Accelerated Aging Models**	D-galactose-treated mouse	Excess galactose metabolism (chemical induction)	ROS overproduction, AGE–RAGE signaling, mitochondrial dysfunction & DDR activation	Systemic aging phenotypes in brain, heart, liver, kidney, reproductive system. Strong DDR activation and apoptosis in multiple tissues.	[108,109]
	Total Body Irradiation (TBI)	DSBs (ionizing radiation)	Ionizing radiation-induced DSBs and ROS leading to DDR activation	Hematopoietic stem cell senescence with long-term marrow impairment, intestinal senescence with dysbiosis and absorption defects, hair whitening. Persistent DDR signaling accelerates senescence.	[110,111]

**Table 3:** Therapeutic strategies targeting the DDR–aging axis and age-related decline.

Category	Mechanism/Target	Representative Agents	Principal Effects
**Direct DDR checkpoint and repair modulation**	Checkpoint inhibition (p21, PUMA, EXO1, p16INK4a), ATM/ATR inhibition	Experimental checkpoint gene suppression; ATM/ATR inhibitors (oncology)	Extends lifespan/tissue maintenance in telomere-deficient mice; ATR restores origin firing in aged liver but induces inflammation without proliferative rejuvenation; human efficacy unproven.
	NAD^+^ restoration (supports PARPs, sirtuins, DNA repair)	NR, NMN	Enhanced DNA repair, mitochondrial function, stress resistance
	Sirtuin activation	Resveratrol, SRT1720, SRT2104	Improved metabolic & inflammatory markers; lifespan extension inconsistent
	Epigenetic modulation (HDAC/DNMT/BET)	Vorinostat, panobinostat; azacitidine, decitabine; JQ1	Restoration of repair fidelity, epigenome remodeling; toxicity limits use.
**Downstream targeting of DDR-induced senescence**	Senolytics (eliminate senescent cells)	Dasatinib + quercetin, navitoclax, fisetin	Reduced senescent cell burden, alleviated dysfunction, lifespan extension in mice
	Senomorphics (suppress SASP, modulate phenotype)	Rapamycin, metformin, ruxolitinib, NF-κB and p38 inhibitors	SASP suppression, reduced inflammation, tissue homeostasis, lifespan extension in models
	Stem cell–based regenerative therapies	MSC/HSC transplantation; partial reprogramming	Restored regenerative capacity, rejuvenation of aged tissues in preclinical models
**Indirect reduction of DNA damage & stress**	Antioxidants, ROS modulators	N-acetylcysteine (NAC), MitoQ, CoQ10, SkQ1	Reduced oxidative stress, DNA damage, delayed senescence
	Dietary antioxidants (Micronutrients)	Vitamins A, C, D, E, K, B-complex; selenium; zinc	Reduction of oxidative DNA damage; maintenance of genomic stability
	Nutritional/metabolic interventions	Caloric restriction, fasting-mimicking diets	Lower ROS production, enhanced genome maintenance, lifespan extension
	Polyphenol-rich nutritional interventions	Resveratrol, quercetin, curcumin (Mediterranean diet pattern)	Enhanced DNA repair and reinforced cellular stress resilience
	Anti-inflammatory strategies	Anakinra (IL-1), tocilizumab (IL-6)	Improved hematopoietic regeneration, tissue homeostasis
	NRF2 activation	Sulforaphane, dimethyl fumarate, bardoxolone	Enhanced stress resilience, genome stability, delayed senescence

## Abbreviations

The following abbreviations are used in this manuscript:

ATM	Ataxia–Telangiectasia Mutated kinase
ATR	Ataxia–Telangiectasia and Rad3-related kinase
BER	Base excision repair
BRCA	Breast cancer susceptibility protein
DDR	DNA damage response
DSB	Double-strand break
dHJ	Double Holliday junction
DNA-PKcs	DNA-dependent protein kinase catalytic subunit
ERCC	Excision repair cross-complementation group
EXO1	Exonuclease 1
FA	Fanconi anemia
FANCM	Fanconi anemia complementation group M protein
HhH2	Helix–hairpin–helix domain
HR	Homologous recombination
HRDC	Helicase and RNase D C-terminal domain
MRN	MRE11–RAD50–NBS1 complex
MRE11	Meiotic recombination 11 protein
MSC	Mesenchymal stem cell
mtDNA	Mitochondrial DNA
NER	Nucleotide excision repair
NHEJ	Non-homologous end joining
NSC	Neural stem cell
PCNA	Proliferating cell nuclear antigen
PD-(D/E)XK	Conserved catalytic motif of nuclease family
POLG	DNA polymerase γ
RPA	Replication protein A
RQC	RecQ C-terminal domain
RTEL1	Regulator of telomere elongation helicase 1
SASP	Senescence-associated secretory phenotype
SF1–SF6	Helicase superfamilies 1–6
TFIIH	Transcription factor IIH complex
WRN	Werner syndrome helicase
XPA	Xeroderma Pigmentosum group A protein
XPB	Xeroderma Pigmentosum group B helicase
XPC	Xeroderma Pigmentosum group C protein
XPD	Xeroderma Pigmentosum group D helicase
XPF	Xeroderma Pigmentosum group F endonuclease (ERCC4)
XPG	Xeroderma Pigmentosum group G endonuclease (ERCC5)
